# Strength in Adhesion: A Multi-Mechanics Review Covering Tensile, Shear, Fracture, Fatigue, Creep, and Impact Behavior of Polymer Bonding in Composites

**DOI:** 10.3390/polym17192600

**Published:** 2025-09-25

**Authors:** Murat Demiral

**Affiliations:** College of Engineering and Technology, American University of the Middle East, Egaila 54200, Kuwait; murat.demiral@aum.edu.kw

**Keywords:** adhesive bonding, polymer adhesives, composite structures, mechanical behavior, structural integrity, fracture toughness, fatigue, viscoelasticity

## Abstract

The growing demand for lightweight and reliable structures across aerospace, automotive, marine, and civil engineering has driven significant advances in polymer adhesive technology. These materials serve dual roles, functioning as matrices in composites and as structural bonding agents, where they must balance strength, toughness, durability, and sometimes sustainability. Recent review efforts have greatly enriched understanding, yet most approach the topic from specialized angles—whether emphasizing nanoscale toughening, multifunctional formulations, sustainable alternatives, or microscopic failure processes in bonded joints. While such perspectives provide valuable insights, they often remain fragmented, leaving open questions about how nanoscale mechanisms translate into macroscopic reliability, how durability evolves under realistic service conditions, and how mechanical responses interact across different loading modes. To address this, the present review consolidates knowledge on the performance of polymer adhesives under tension, shear, fracture, fatigue, creep, and impact. By integrating experimental findings with computational modeling and emerging data-driven approaches, it situates localized mechanisms within a broader structure–performance framework. This unified perspective not only highlights persistent gaps—such as predictive modeling of complex failure, scalability of nanomodified systems, and long-term durability under coupled environments—but also outlines strategies for developing next-generation adhesives capable of delivering reliable, high-performance bonding solutions for demanding applications.

## 1. Introduction

Polymer adhesives are central to composite structures, serving both as matrices in fiber-reinforced composites and as bonding agents in structural joints. Their ability to provide robust adhesion, transfer loads, and resist environmental degradation has made them indispensable in aerospace, automotive, marine, and civil engineering. Advances in adhesive technologies have enabled lightweight, high-performance structures that meet increasingly stringent durability requirements. Beyond traditional functions, modern adhesives are being tailored for multifunctional roles such as damage sensing, vibration damping, and self-healing, broadening their relevance in next-generation systems.

**Figure 1:**
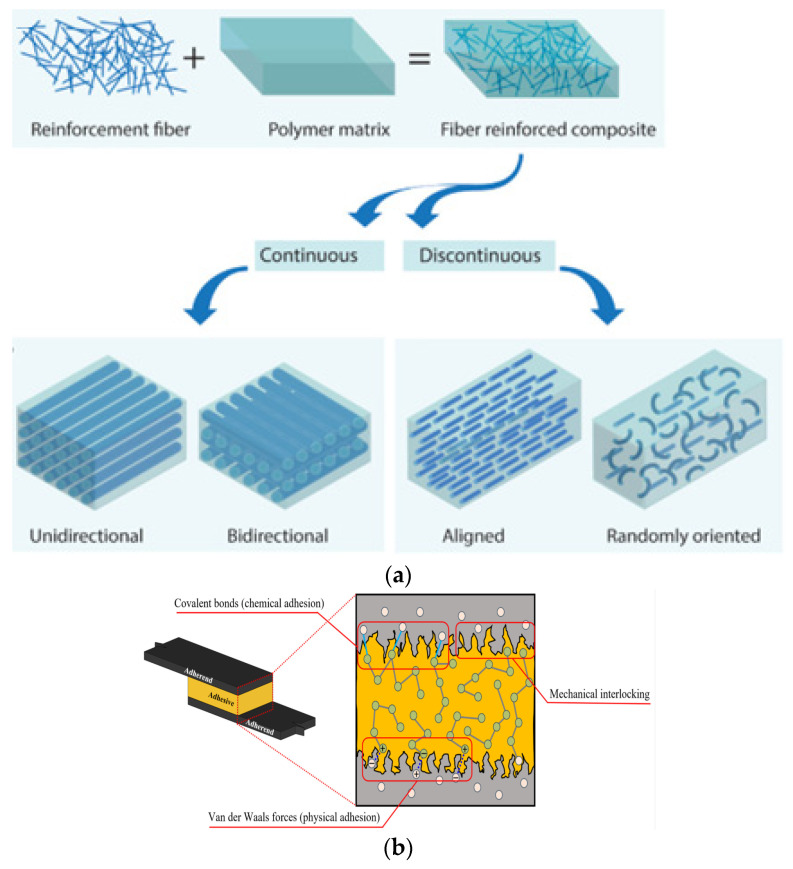
The general structural assembly of fiber reinforced plastic composites and their classification (**a**) [2], bonding mechanism using adhesive in a single lap joint (**b**) [3].

As matrices, adhesives encapsulate and bind reinforcing fibers, enabling effective stress transfer, toughness enhancement, and environmental protection (Figure 1a). Thermosetting resins such as epoxies and polyurethanes dominate, though their brittleness drives research into hybrid matrices with thermoplastics or nanofillers (e.g., graphene, CNTs) to improve ductility, crack resistance, and impact tolerance. When used as bonding agents (Figure 1b), adhesives eliminate mechanical fasteners, reduce stress concentrations, and improve fatigue resistance and sealing performance in composite assemblies [1]. These dual roles make them critical to structural reliability in dynamic service conditions.

Understanding adhesive behavior relies on both experimental and computational approaches. Classical tests such as lap shear, Double Cantilever Beam (DCB), and End-Notch Flexure (ENF) provide baseline strength and toughness measures, while fatigue rigs, creep frames, and impact tests capture long-term and dynamic responses. Advanced tools such as Digital Image Correlation (DIC) and Scanning Electron Microscope (SEM) offer insights into strain localization and fracture mechanisms. Computationally, finite element analysis (FEA) and cohesive zone modeling (CZM) have been widely applied to predict stress distributions and crack growth, with recent multiscale models linking molecular architecture to macroscopic behavior. Machine learning is emerging as a powerful tool to accelerate adhesive design by exploiting combined experimental and simulation datasets. Despite these advances, challenges persist. Adhesives rarely experience purely tensile or shear stresses; real structures impose mixed-mode loading, often leading to combined cohesive and interfacial failures. Similarly, fatigue, creep, and high-strain-rate conditions introduce complex degradation pathways that remain insufficiently understood. Environmental durability adds further uncertainty, as moisture, ultraviolet (UV) radiation, and temperature cycling accelerate chemical and physical degradation. Moreover, achieving consistent bond quality in large-scale assemblies is hindered by variability in surface preparation, curing protocols, and process control [4].

Several review papers have recently contributed to advancing the field, though most approach the subject from specific angles rather than holistically. For instance, ref. [5] emphasizes nanoscale toughening mechanisms such as crack deflection and particle bridging, while Mousavi et al. [6] examined carbonaceous nanofillers in epoxy adhesives, showing notable gains in fracture toughness through stress redistribution and energy dissipation. Similarly, the article [7] consolidated advances in multifunctional adhesives, but pointed out unresolved issues related to long-term durability. Complementing these synthetic approaches, the review on high-performance bio-based wood adhesives [8] underscored the potential of sustainable formulations but noted persistent trade-offs between eco-friendliness and mechanical robustness. Finally, the paper [9] shed light on the microstructural origins of joint failure, linking interfacial flaws and micro-cracking to structural-scale degradation. These works collectively highlight remarkable progress in nanoscale design, sustainability, and failure mechanisms, but they also reveal gaps in connecting these isolated findings to a unified understanding of adhesive performance across real service conditions.

Although these reviews provide valuable insights into fracture mechanisms, material design strategies, and microscopic failure processes, they remain constrained to specific dimensions of adhesive science. Most focus either on nanocomposite toughening, eco-friendly formulations, or microscopic damage mechanisms, leaving a gap in comprehensive treatments that span all mechanical properties under diverse loading modes. The present study addresses this limitation by consolidating the mechanical response of polymer adhesives across tensile, shear, fracture toughness, fatigue, creep, and high-strain-rate conditions. By integrating experimental evidence, numerical modeling, and emerging approaches such as machine learning, this review situates nanoscale and microstructural insights within a broader structure–performance context, offering a more complete perspective on designing reliable bonded structures for demanding applications [10].

Emerging strategies aim to overcome current limitations. Nano-enhanced adhesives incorporating carbon nanotubes, graphene, or silica improve strength and thermal stability, though scalability remains a challenge. Self-healing systems show promise for extending service life, while bio-based formulations offer sustainable alternatives to petroleum-derived products [11]. There is also growing interest in adhesives engineered for extreme conditions, including cryogenic temperatures and high strain rates, where conventional systems often fail. However, gaps remain in translating laboratory findings into predictive models for real-world performance, especially under multiaxial and service-specific loading.

In summary, this review focuses explicitly on the mechanical behavior of polymer adhesives under diverse loading scenarios. It synthesizes insights from tensile and shear strength, fracture toughness, fatigue, creep, and high-strain-rate behavior, integrating experimental data, numerical modeling, and emerging research directions. By bridging molecular-scale mechanisms (e.g., crosslink density, filler dispersion) with structural-scale performance, the paper provides a more integrated perspective for designing durable, high-performance adhesive joints.

The literature surveyed was identified using Web of Science, Scopus, and Google Scholar, focusing primarily on 2020–2025 publications, with seminal earlier works included for context. Search terms combined adhesive types with performance-related keywords (tensile, shear, fatigue, impact, creep). Studies were included if they provided mechanical characterization, failure analysis, or modeling in structural or composite applications, with priority given to peer-reviewed sources. Excluded were works limited to cost analysis, analytical works, insufficient methodology, or derivative publications. This strategy ensured broad coverage of current knowledge while maintaining a clear focus on mechanical performance in structural contexts.

## 2. Overview of Polymer Adhesives and Characterization of Mechanical Properties

### 2.1. Overview of Polymer Adhesives in Composites and Bonded Joints

#### 2.1.1. Types of Adhesives Used

**Figure 2:**
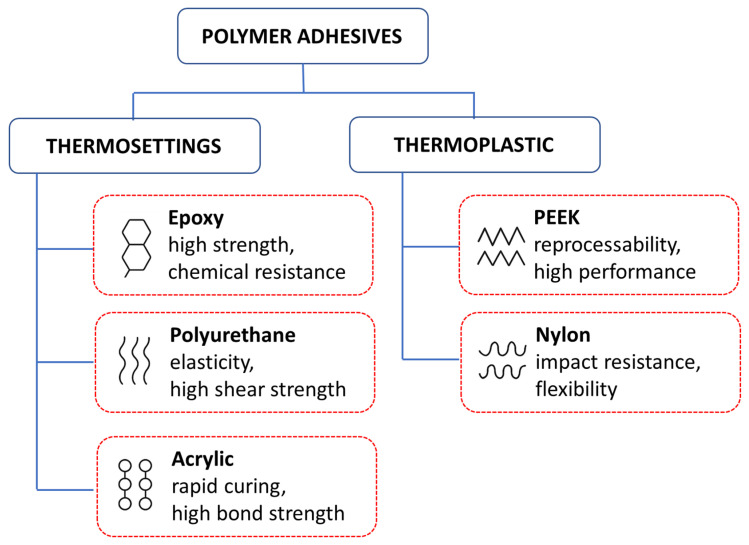
Classification and key characteristics of polymer adhesives.

Polymer adhesives are fundamental to the performance and longevity of composite structures, functioning as both matrices in fiber-reinforced composites and as bonding agents in structural joints. They are broadly categorized into thermosetting and thermoplastic types, each offering distinct properties tailored to specific engineering applications (Figure 2).

Thermosetting adhesives—including epoxy, polyurethane, and acrylic systems—are widely used due to their high strength and durability. Upon curing, they form cross-linked networks that provide superior chemical resistance and long-term stability.

- Epoxy adhesives are extensively applied in aerospace, automotive, and construction because of their stiffness, adhesion to metals, ceramics, and composites, and robustness under environmental exposure. Their brittleness, however, limits impact performance, prompting modifications such as rubber or thermoplastic toughening [12]. More recently, formulations designed to maintain performance across wide temperature ranges have expanded their use in extreme environments [13].
- Polyurethane adhesives offer a balance of elasticity and high shear strength, making them suitable for flexible adherends and vibration-damping applications. Their fatigue resistance and toughness make them advantageous where epoxy’s brittleness is problematic. Recent studies on dynamic polymer networks demonstrate potential for recyclable, reprocessable polyurethane adhesives by enabling bond-exchange reactions, though industrial scalability remains a challenge [14].
- Acrylic adhesives combine rapid curing, high bond strength, and ease of processing, which make them attractive in high-volume, cost-sensitive applications such as automotive assembly. Thermosetting acrylics can withstand elevated temperatures and aggressive environments, though they often exhibit lower long-term fatigue resistance than polyurethanes [15]. Cyanoacrylate adhesives cure instantly and bond diverse substrates, from metals and ceramics to biological tissues. However, their brittleness and poor peel/impact resistance limit structural use, confining them to small-area, temporary, or niche applications.

Thermoplastic adhesives offer advantages in reprocessability and toughness. Unlike thermosets, they can be reheated and reshaped, facilitating repair and recycling.

- Polyether ether ketone (PEEK) adhesives combine mechanical strength, chemical resistance, and high-temperature performance. Their dimensional stability and lightweight nature make them suitable for aerospace and electronics, while recent work highlights their potential in biomedical applications due to biocompatibility [16].
- Nylon-based adhesives provide flexibility and impact resistance, performing well in dynamic load conditions. Hot-melt nylon systems show favorable peel strength and fast setting, supporting their use in industrial applications where toughness and wear resistance are essential [17].

Ultimately, the choice between thermosetting and thermoplastic adhesives depends on application-specific requirements such as load-bearing capacity, fatigue resistance, environmental durability, and end-of-life considerations (e.g., recyclability). While thermosets remain dominant in structural bonding due to their stiffness and thermal stability, thermoplastics are increasingly gaining attention for sustainable and repairable systems.

#### 2.1.2. Adhesion Mechanisms in Different Applications

The effectiveness of polymer adhesives in composite structures is largely dictated by the adhesion mechanisms that enable bonding, which differ depending on whether the adhesive acts as a matrix in fiber-reinforced composites or as a bonding agent in joints. A clear understanding of these mechanisms is essential for optimizing strength, durability, and long-term reliability. An overview of key interfacial interactions is summarized in Table 1.

When used as a matrix in fiber-reinforced composites, adhesion arises primarily through chemical bonding and fiber–matrix interactions. Efficient stress transfer depends strongly on chemical compatibility between the polymer and fiber surface. Functional groups such as hydroxyl, carboxyl, or amino moieties promote covalent or hydrogen bonding with reactive polymer chains. Surface modification techniques, including silane coupling agents, establish molecular bridges that significantly enhance interfacial strength [18]. Likewise, plasma-treated or oxidized fibers improve wettability and adhesion, yielding superior mechanical performance [19].

Mechanical interlocking also contributes to fiber–matrix adhesion when polymer resins infiltrate surface asperities or pores, thereby anchoring fibers within the matrix. Treatments such as grit blasting or chemical etching increase surface roughness and available bonding area, enhancing load transfer [20]. The effectiveness of interlocking depends on adhesive viscosity, processing conditions, and surface topography.

**Table 1:** Summary of adhesion mechanisms of polymer adhesives in composite structures.

Adhesive Role	Primary Mechanisms	Key Factors and Surface Treatments	Effects on Performance	References
Matrix in Fiber-Reinforced Composites	- Chemical bonding (covalent or hydrogen bonds with fiber surface groups) - Mechanical interlocking (infiltration of surface asperities)	- Functional groups on fiber surface (e.g., –OH, –COOH, –NH_2_) - Use of silane coupling agents for molecular bridging - Surface modifications: plasma treatment, oxidation, grit blasting, chemical etching	- Enhanced stress transfer and interfacial strength - Improved wettability and adhesion strength - Superior mechanical performance of composites	[12,18,20]
Bonding Agent in Adhesive Joints	- Surface adhesion (chemical compatibility and surface energy) - Mechanical interlocking (penetration into microscopic irregularities) - Van der Waals forces (secondary molecular attractions)	- Surface treatments: mechanical abrasion, chemical etching, plasma treatment - Laser ablation and nano-patterning for increased anchoring - Surface energy matching between adhesive and substrate	- Improved wetting and adhesion - Enhanced joint strength, especially in composite-to-metal bonds - Complementary van der Waals forces improving durability	[21,22,23,24]
Advanced Approaches	- Nano-reinforced adhesives (graphene oxide, carbon nanotubes)	- Nanoparticle incorporation to enhance bonding and fracture toughness	- Increased interfacial bonding and resistance to environmental degradation - Promising for next-generation composite structures	[25]

In bonded joints, adhesion mechanisms differ from composite matrices but still rely on a combination of surface adhesion, mechanical interlocking, and secondary interactions. Surface adhesion is strongly influenced by substrate compatibility and wetting behavior. Adhesives with higher surface energy generally form stronger bonds with high-energy substrates [21]. Pre-bond treatments—including abrasion, chemical etching, and plasma treatment—remove weak boundary layers and increase wettability, thus improving interfacial strength. Plasma treatment is particularly attractive because it modifies surface chemistry without introducing structural damage [22].

Mechanical interlocking in joints follows the same principle as in fiber–matrix systems but is especially critical in dissimilar material bonding (e.g., composite-to-metal), where thermal expansion and stiffness mismatches can compromise durability [26]. Advanced techniques such as laser ablation and nano-patterning have been explored to optimize interfacial texturing, leading to stronger and more reliable adhesion [23].

Van der Waals forces, though relatively weak, provide additional molecular-level attraction between the adhesive and substrate. Secondary interactions, such as dipole–dipole and dispersion forces, can improve wettability and complement stronger chemical and mechanical mechanisms [24]. However, relying solely on these weak forces is insufficient for structural applications.

Overall, the interplay of chemical bonding, mechanical interlocking, and tailored surface treatments determines the performance and longevity of adhesive bonds. Recent progress in nanotechnology has further advanced adhesion strategies: nano-reinforced adhesives incorporating fillers like graphene oxide or carbon nanotubes show enhanced interfacial bonding, fracture toughness, and environmental resistance [25]. Despite these advances, a gap remains in translating laboratory-scale nano-modification strategies into scalable, cost-effective industrial practices. Future work should address durability under service conditions and the balance between manufacturing feasibility and improved performance.

#### 2.1.3. Factors Affecting Adhesive Performance

The mechanical performance and durability of polymer adhesives in composite structures are strongly influenced by both manufacturing parameters and environmental exposure. Among the manufacturing factors, curing conditions are particularly critical for thermosetting adhesives. The curing process involves chemical crosslinking, which defines the adhesive’s stiffness, glass transition temperature (Tg), and chemical resistance. Crosslink density at the molecular scale governs macroscopic behavior: higher densities enhance stiffness and thermal stability but often at the expense of toughness and strain-to-failure. Conversely, lower crosslink densities improve ductility but reduce modulus and strength, highlighting the inherent trade-off that must be optimized for each application [27,28]. Inadequate curing can result in incomplete polymerization and poor adhesion, while over-curing may induce brittleness, both of which compromise structural reliability.

Filled adhesive systems add another layer of complexity. The dispersion and interfacial bonding of nanoparticles or microfillers (e.g., silica, graphene oxide, rubber particles) directly affect load transfer efficiency. Well-dispersed fillers improve modulus, fracture toughness, and crack resistance, while poor dispersion can generate stress concentrations that initiate premature failure. Advanced characterization tools such as FTIR, DSC, DMA, and nano-CT are increasingly used to link filler distribution and molecular architecture to bulk mechanical response. Bond-line thickness also plays a decisive role: thinner layers favor efficient load transfer but may introduce stress concentrations, whereas thicker layers improve energy absorption but reduce stiffness and load-bearing capacity [29]. Achieving the right balance between strength and toughness, therefore, requires careful bond-line optimization. Substrate surface treatment is equally vital. Cleaning, roughening, and chemical or plasma treatments increase wettability and introduce functional groups that enhance chemical bonding, while inadequate preparation often leads to interfacial failure under load [30,31].

Beyond processing, environmental factors dominate long-term performance. Moisture absorption, thermal cycling, UV radiation, and chemical exposure can severely degrade adhesion strength, fracture toughness, and fatigue resistance. Humidity ingress plasticizes the polymer network, lowers Tg, and accelerates hydrolytic degradation of groups such as esters and amides. Fracture energy reductions of 30–40% have been reported in epoxy systems under cyclic loading [32]. Standardized test methods are widely employed to simulate service conditions, including hygrothermal aging (ASTM D1183 [33], ISO 9142 [34]), UV weathering (ASTM G154 [35]), thermal cycling, and salt-spray/chemical immersion tests (ASTM B117 [36], ISO 9227 [37]) [38].

Among these exposures, thermal cycling generates residual stresses at dissimilar adherend–adhesive interfaces, promoting microcracking or debonding. Even toughened epoxies may lose shear strength under repeated cycles, while flexible polyurethanes retain comparatively better performance. UV exposure induces chain scission and radical formation, leading to chalking, embrittlement, and reduced toughness in systems such as epoxies and acrylics, unless stabilizers or protective coatings are applied [39]. Chemical agents—including fuels, lubricants, and salts—can penetrate the adhesive network, inducing swelling, plasticization, or chemical attack. Aerospace and marine studies have linked hydraulic fluid and salt exposure to significant reductions in shear strength and fatigue life. Although high-performance thermoplastics such as PEEK adhesives offer superior resistance, they impose higher material costs [40].

To mitigate these degradation pathways, modern adhesive formulations incorporate nano-fillers, UV stabilizers, hydrophobic agents, and barrier coatings. While these strategies enhance durability, they may also alter viscosity, curing kinetics, or processability, underscoring the trade-offs in adhesive design and highlighting the need for further research into formulations that balance durability with manufacturability [41,42].

### 2.2. Characterizing Mechanical Properties

#### 2.2.1. Experimental Methods

The mechanical properties of polymer adhesives in composite structures are evaluated using a range of experimental methods. These methods help assess the strength, toughness, fatigue resistance, and failure mechanisms of adhesives under different loading conditions. Mechanical testing approaches, fracture and fatigue testing methods, and microscopic and spectroscopic failure analysis techniques provide valuable insights into the performance and durability of polymer adhesives. The selection of an appropriate characterization technique depends on the specific mechanical property being investigated and the conditions under which the adhesive is expected to perform. Table 2 presents a summary of mechanical, fracture, fatigue, and failure analysis methods for polymer adhesives in composite structures.

#### 2.2.2. Numerical Modeling and Simulation Approaches

Numerical modeling and simulation techniques are essential for analyzing the mechanical behavior of polymer adhesives within composite structures. These methods offer a detailed understanding of stress distribution, failure modes, and durability under different loading scenarios. Approaches such as cohesive zone modeling (CZM), finite element analysis (FEA), and molecular dynamics (MD) simulations allow for accurate prediction of adhesive behavior and support the optimization of joint configurations. Through multi-scale modeling, molecular-level interactions can be connected to bulk mechanical properties, enhancing the reliability and efficiency of adhesive applications in structural systems.

##### Cohesive Zone Models for Fracture Prediction

Cohesive zone models (CZM) are extensively used to predict fracture behavior in polymer adhesives, providing a computational framework for simulating crack initiation and propagation. These models define a traction–separation law at the adhesive interface, allowing interfacial failure to be described in terms of cohesive parameters such as fracture toughness, adhesive strength, and energy dissipation. CZM is particularly effective in studying failure mechanisms in bonded joints and fiber–matrix interfaces, making it a critical tool for analyzing adhesive performance in structural applications [68]. When incorporated into finite element frameworks, CZM enables the precise prediction of debonding behavior under Mode I (opening), Mode II (shearing), and mixed-mode fracture. This approach facilitates detailed simulations of crack growth and interfacial failure, allowing engineers to assess stress distributions and energy dissipation mechanisms that are central to designing durable and damage-resistant adhesive joints [69].

CZM captures the elastic response up to the peak load, followed by the initiation of damage and progressive crack propagation. The constitutive basis is typically a traction–separation law that links cohesive tractions (normal and shear stresses) to the relative displacements between nodes of cohesive elements. This allows the simulation of the elastic response up to the peak stress values ($t_{n}^{0}$ (tension) or $t_{s}^{0}$ (shear)), followed by a gradual stiffness reduction as material degradation advances until complete failure. The energy dissipated during this process corresponds to the areas under the traction–separation curves in tension ($G_{n}$) and shear ($G_{s}$), with maximum relative displacements ($\delta_{n}^{f}$, $\delta_{s}^{f}$) defined by satisfying critical energy release conditions. Regardless of the CZM shape, the initial linear–elastic response is governed by a constitutive stiffness matrix that relates tensile and shear stresses across the interface.

**Figure 3:**
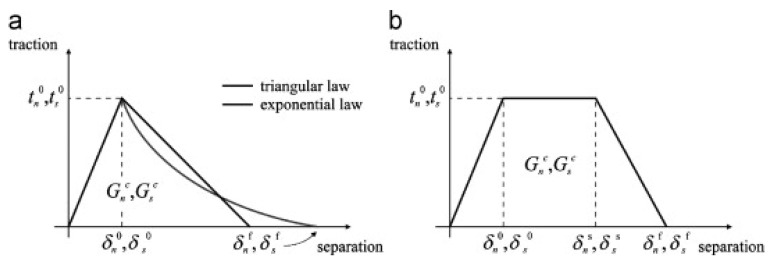
CZM formulations featuring triangular, exponential (**a**), and trapezoidal (**b**) traction–separation profiles. Reprinted from Ref. [70] with permission from Elsevier, 2013.

In this review, three CZM shapes—triangular, linear-exponential, and trapezoidal—are discussed and schematically illustrated in Figure 3. The triangular CZM is the simplest and most widely adopted form, characterized by a linear loading branch up to the peak traction ($t_{n}^{0}$ (tension) or $t_{s}^{0}$ (shear)), followed by a linear softening branch until complete separation. Its computational efficiency makes it suitable for brittle or nearly brittle interfaces, but the sharp stress drop may underpredict energy dissipation in ductile adhesive joints. The linear-exponential law, by contrast, exhibits a linear increase up to peak traction, followed by an exponential softening phase until failure. This shape better captures progressive damage accumulation by maintaining finite traction at larger separations, thereby representing ductile adhesives more realistically. It can be regarded as an approximation of the full-exponential law, but with a sharper stress drop compared to the triangular law.

The trapezoidal CZM law introduces three distinct stages: (i) a linear elastic loading phase, (ii) a plateau region at constant traction ($t_{n}^{0}$ (tension) or $t_{s}^{0}$ (shear)) over a finite separation range, and (iii) a linear softening phase until full decohesion. The plateau phase is particularly advantageous for modeling plastic deformation or yielding at the interface, as seen in toughened adhesives or composite interfaces exhibiting fiber bridging. Trapezoidal laws generally dissipate more energy than triangular or exponential profiles for the same peak traction, leading to improved predictions of joint toughness and resistance to unstable crack growth. Overall, the choice of CZM shape significantly influences predicted fracture energy, crack initiation force, and overall joint response: triangular laws are best suited for brittle failure and efficiency, exponential laws for ductile progressive damage, and trapezoidal laws for interfaces with extended plasticity.

In fiber-reinforced composites, CZM plays a central role in simulating fiber–matrix debonding and crack-bridging effects. Although polymer adhesives primarily act as load-transfer media between adherends rather than bulk matrices, their fracture behavior directly affects joint performance and structural integrity. In certain manufacturing processes, such as co-curing or co-bonding, film adhesives may locally contribute to the matrix, but their principal role is the transfer of stresses and resistance to interfacial failure. Accurate modeling of these mechanisms is essential, as interfacial debonding often governs delamination growth and long-term reliability. To enhance predictive accuracy, CZM is increasingly integrated with experimental techniques such as digital image correlation and acoustic emission analysis, which provide complementary information on strain fields and crack evolution. Such hybrid approaches strengthen the link between numerical predictions and real-world adhesive performance, offering valuable insights for optimizing bonding strategies in advanced composite structures [71].

##### Finite Element Analysis (FEA) of Adhesive Joints and Matrices

Finite element analysis (FEA) is a widely utilized computational technique for investigating stress distribution, deformation, and failure mechanisms in adhesive joints and composite matrices. By discretizing adhesive structures into finite elements, FEA solves governing equations to predict mechanical responses under applied loads. This approach enables the assessment of adhesive joint performance in terms of load-transfer efficiency, stiffness, and failure modes, making it an essential tool for structural integrity analysis [72]. In bonded joints, FEA provides detailed insight into stress distribution across the adhesive layer, identifying regions prone to failure. The incorporation of nonlinear material models further accounts for the viscoelastic and plastic behavior of polymer adhesives, offering a more realistic representation of their response. Additionally, parametric studies using FEA allow the evaluation of factors such as bond-line thickness, adhesive type, and surface treatment, thereby guiding optimization of joints for enhanced durability and strength.

**Figure 4:**
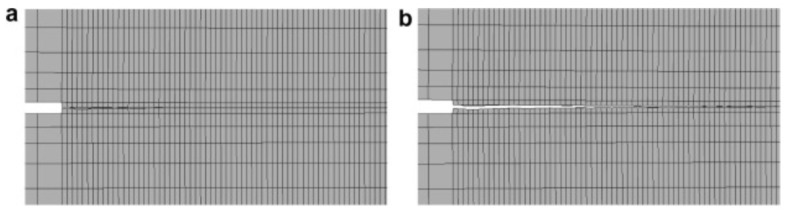
Crack propagation modeled using the XFEM algorithm begins at the crack tip (**a**) and progresses horizontally along the adhesive bondline (**b**). Reprinted from Ref [75] with permission from Elsevier, 2011.

Recent advances have expanded FEA capabilities, with the Extended Finite Element Method (XFEM) providing a powerful framework to model crack initiation and propagation without predefined crack paths (see Figure 4). In XFEM-based simulations, cracks are initialized at stress concentration points (e.g., notch tips) and propagate along adhesive bondlines when the traction–separation criterion is met. By enriching the displacement field near the crack tip with additional shape functions, XFEM captures discontinuities within existing elements, avoiding remeshing and improving efficiency. This feature is particularly advantageous in adhesive joints, where cracks typically remain constrained within the adhesive layer and may evolve under mixed-mode (Mode I and Mode II) loading [73,74]. Crack initiation is often governed by a maximum principal stress criterion, while propagation follows the computed direction of maximum energy release rate. This approach captures interfacial debonding behavior, stiffness degradation, and unstable fracture, thereby enabling an accurate prediction of load–displacement responses and the ultimate joint capacity. Compared to traditional CZM with predefined paths, XFEM provides greater flexibility in simulating progressive fracture development.

For polymer adhesives acting as composite matrices, FEA also plays a key role in examining fiber–matrix interactions, including stress–strain behavior, thermal expansion mismatches, and fiber debonding. Multi-scale FEA approaches further bridge microstructural features with macroscopic performance, enabling predictions that integrate thermal, mechanical, and environmental effects on composite behavior. Such modeling has become indispensable in designing advanced adhesives with improved toughness, reliability, and application-specific performance [76].

A critical comparison of FEA and CZM highlights their respective strengths and limitations under complex, mixed-mode loading. FEA offers a flexible framework for analyzing stress distributions and global structural response, but prediction accuracy can be strongly affected by mesh refinement and assumed material models. In contrast, CZM excels in simulating interfacial fracture processes by explicitly representing crack initiation and propagation. Adediran et al. [77] demonstrated that different adhesive model representations within FEA produced noticeably different lap joint strength predictions, emphasizing the importance of experimental calibration. Similarly, recent work [78] showed that CZM-based approaches more closely replicated experimental failure paths under varied loading rates compared to continuum FEA. Together, these findings suggest that hybrid approaches, which integrate the global evaluation capability of FEA with the local fracture fidelity of CZM, may offer the most effective balance between computational efficiency and predictive reliability.

##### Multi-Scale Modeling of Adhesives in Composite Laminates

Multi-scale modeling techniques integrate different length scales—from molecular-level interactions to macrostructural performance—providing a comprehensive framework for understanding polymer adhesive behavior [79]. In composite laminates, adhesives contribute at the micro-scale through fiber–matrix interactions and at the macro-scale by maintaining bond-line integrity in joints. These approaches enable researchers to capture the influence of microstructural features, such as voids, defects, and molecular alignment, on the overall mechanical performance of adhesive joints [80]. However, despite their promise, the coupling between scales is often non-trivial, and the transfer of parameters across different length scales remains a significant challenge.

**Figure 5:**
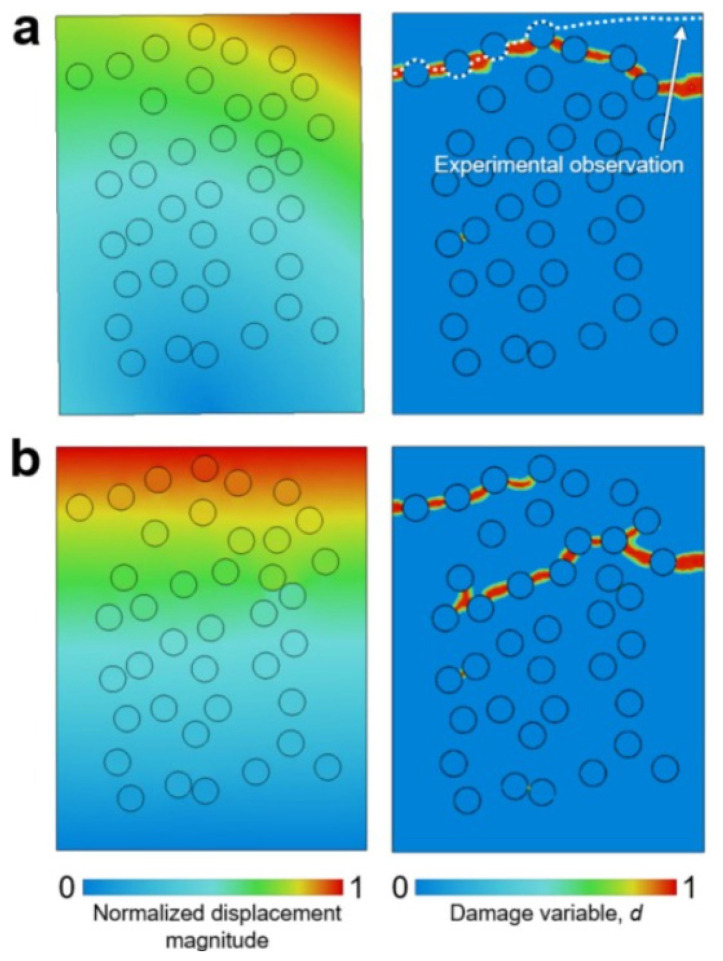
Displacement distributions (left) and matrix crack trajectories (right) for RVE-1 under two boundary condition scenarios: (**a**) boundary conditions obtained from DIC measurements and (**b**) simplified conditions applied as a uniform vertical displacement along the domain’s top edge. Reprinted from Ref [82] with permission from Elsevier, 2025.

At the micro-scale, representative volume elements (RVEs) are widely used to simulate fiber–matrix interactions, capturing the effects of fiber orientation, interfacial bonding, and damage progression. Figure 5 illustrates results for RVE-1: the left panel shows displacement distributions, while the right depicts crack trajectories under two boundary condition scenarios. Realistic conditions extracted from DIC measurements reproduce heterogeneous deformation fields observed experimentally, leading to crack paths that closely match tests. By contrast, simplified uniform displacement boundary conditions yield more homogeneous deformation and altered crack trajectories, potentially underestimating local stress concentrations. This highlights not only the critical role of realistic boundary conditions but also the sensitivity of model predictions to assumptions—an aspect that can limit generalizability across loading scenarios.

The integration of such micro-scale results into macro-scale finite element analysis (FEA) models enhances predictive reliability by embedding heterogeneity into structural-level assessments [81]. Yet, uncertainties remain regarding how well these upscaled inputs capture damage evolution under complex loading, particularly in mixed-mode or fatigue conditions. While multiscale frameworks improve failure prediction, their computational intensity and limited experimental validation across diverse adhesive systems restrict their current industrial application.

Beyond structural analysis, multi-scale modeling has also been employed for adhesive design, linking chemical composition and curing conditions with macroscopic performance. Molecular dynamics simulations coupled with continuum-level FEA offer insights into how formulation parameters influence toughness, durability, and environmental resistance [83]. Nevertheless, discrepancies often arise between molecular-level predictions and bulk adhesive performance, especially under long-term service conditions where environmental degradation and interfacial aging play critical roles. Addressing these gaps through broader validation studies and more efficient multiscale coupling strategies remains essential for translating modeling advances into practical adhesive design.

##### Molecular Dynamics (MD) Simulations

**Figure 6:**
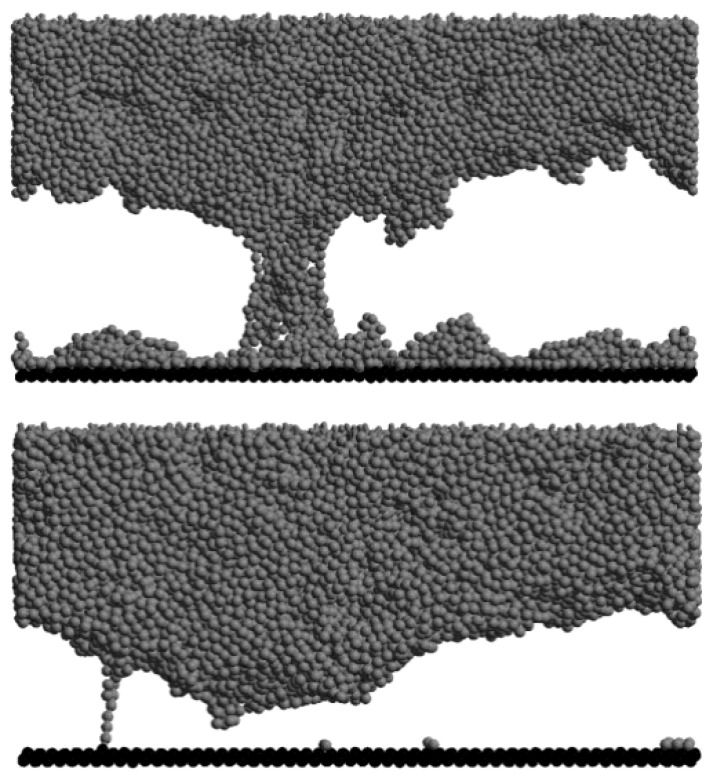
Molecular dynamics simulation outcomes showing cohesive failure at the top and adhesive failure at the bottom in epoxy materials. Reprinted from Ref [85] with permission from American Chemical Society, 2024.

Molecular dynamics (MD) simulations provide atomistic-level insights into polymer adhesive behavior by explicitly modeling interactions between individual molecules. Figure 6 illustrates typical outcomes for epoxy systems: the top panel shows a cohesive failure mode, where fracture occurs within the bulk adhesive, leaving material on both sides of the joint; the bottom panel shows an adhesive failure mode, where debonding occurs at the interface, leaving one surface clean. These contrasting outcomes highlight how fracture pathways are determined by the balance between bulk network strength and interfacial bonding.

In MD studies, controlled separation or shear displacements are applied at the molecular scale, enabling the direct observation of bond-breaking events in real time. By employing accurate force fields, MD simulations not only capture these atomic-scale fracture mechanisms but also allow the calculation of material properties such as elastic modulus, fracture energy, and adhesion strength. Comparisons between cohesive and adhesive failures reveal how interfacial chemistry, crosslink density, or surface treatments govern fracture behavior—providing mechanistic explanations that are often difficult to isolate experimentally [84].

Beyond identifying failure modes, MD simulations clarify the molecular origins of adhesion by quantifying van der Waals interactions, hydrogen bonding, and covalent crosslinking at adhesive–substrate interfaces. Such insights are critical for the rational design of adhesives, as they allow researchers to tailor molecular composition to favor desirable outcomes (e.g., cohesive rather than adhesive failure) and to improve compatibility with different substrates. In silico screening of modified molecular structures has been used to predict formulations with improved adhesion strength and greater resistance to environmental degradation mechanisms such as moisture uptake, thermal cycling, and chemical attack [86].

A key advancement has been the integration of MD simulations with continuum-scale methods such as finite element analysis (FEA), creating multi-scale frameworks that connect atomistic interactions with macroscopic performance. This approach bridges the gap between nanoscale failure events and structural-scale mechanics, significantly improving the predictive accuracy of adhesive joint models. Such multi-scale modeling is particularly relevant for aerospace, automotive, and civil engineering applications, where failure prevention and long-term structural integrity are paramount [87].

## 3. Mechanical Properties

The mechanical properties of polymer adhesives are essential for ensuring the structural integrity and long-term performance of composite materials and bonded joints. These adhesives must endure various loading conditions, including tensile, shear, impact, fatigue, and creep, to maintain durability and reliability in engineering applications. A thorough understanding of the mechanical response of adhesives under different stress states is necessary for optimizing their use in composite structures, improving joint strength, and preventing premature failure.

### 3.1. Tensile and Shear Strength

The stress–strain behavior of polymer adhesives is a fundamental characteristic in evaluating their mechanical performance. The response of adhesives varies depending on whether they function as matrix materials in fiber-reinforced composites or as bonding agents in structural joints [88]. When used as matrices, adhesives must combine stiffness and ductility to enable efficient load transfer to reinforcing fibers. A robust matrix facilitates stress distribution, reducing fiber–matrix debonding and enhancing overall composite strength [26,89]. Conversely, in bonded joints, the adhesive layer serves as the primary medium for transferring loads between substrates. Here, high tensile and shear strength are essential, as the ability to sustain stress without excessive deformation or premature fracture directly determines structural efficiency [90].

Adhesive stress–strain curves typically consist of an initial linear elastic region, followed by nonlinear plastic deformation and, ultimately, failure. Brittle adhesives fracture suddenly with little plasticity, while toughened systems sustain greater elongation, resisting crack propagation more effectively [91]. Load transfer mechanisms also differ by application: in composites, stresses transfer from matrix to fibers through shear at the fiber–matrix interface, while in bonded joints, transmission occurs across the adhesive layer. Failures may initiate via stress concentrations, cohesive cracking, or interfacial debonding [92].

The summarized data in Table 3 highlight tensile and shear strength ranges for five major adhesive families—epoxies, polyurethanes, acrylics, PEEK, and nylon. Epoxies dominate structural applications owing to their high strength, but brittleness remains a persistent limitation. Toughening approaches (rubber particles, thermoplastics, nanofillers) often raise fracture toughness, with graphene- or alumina-modified epoxies surpassing conventional limits. Yet scalability and cost issues restrict widespread adoption. Polyurethanes display far greater variability: conventional grades reach ~14 MPa tensile strength [93], while advanced formulations such as disulfide-exchange or Diels–Alder PUs achieve up to 92 MPa [94,95], rivaling or exceeding epoxy levels. This variability indicates that “polyurethane” is not a uniform category but a design-dependent spectrum. Acrylics combine toughness and processability but show strength ranges (20–55 MPa tensile, 10–25 MPa shear) strongly dependent on curing and substrate, complicating cross-study comparisons. High-performance thermoplastics expand possibilities: PEEK adhesives reach 70–100 MPa in tensile strength and 20–40 MPa in shear strength, though performance hinges on surface treatments; nylon achieves 40–80 MPa tensile strength but remains moisture-sensitive, reducing long-term stability. These ranges underscore the promise of advanced systems while exposing significant heterogeneity due to formulation, processing, and interfacial design.

This variability reveals contradictions across the literature. Polyurethanes, long considered weaker than epoxies, now demonstrate epoxy-level strength when engineered with reversible or hydrogen-bonding crosslinks [94,95]. In contrast, Ribas et al. [93] reported values an order of magnitude lower, showing that formulation dominates outcomes more than adhesive chemistry labels. Epoxies, though generally consistent, remain highly curing-sensitive: extended curing raised tensile strength by 69% and shear strength by 23% [96], proving that processing is a major confounding variable. For PEEK and nylon, surface modification decisively alters adhesion, but standardized reporting is scarce, hindering reproducibility.

Bond-line thickness, adherend geometry, and surface roughness further complicate interpretation across systems. Thin layers improve stiffness and load transfer but concentrate stresses, while thicker layers absorb more energy but reduce rigidity. Rodrigues et al. [97] demonstrated that epoxy–polyurethane hybrids can offset these trade-offs, but optimization is system-specific and sometimes contradictory. This unresolved tension between toughness and stiffness highlights a core design challenge: achieving efficiency under realistic service conditions rather than idealized laboratory setups.

Despite progress, research gaps persist. First, tensile and shear data are rarely generated under identical conditions, complicating direct comparisons across adhesives. Second, most work focuses on static loading, with cyclic, impact, and mixed-mode stresses still underexplored, despite their relevance in aerospace and automotive structures. Third, environmental durability under humidity, thermal cycling, and aging is inconsistently studied—even though these dominate service life. For nylon, moisture uptake remains critically under-characterized. Finally, nanostructured and multifunctional adhesives show short-term promise, but long-term reliability, scalability, and failure mechanisms remain insufficiently documented.

Table 3, therefore, reinforces both the potential and the limitations of current adhesive systems. Future research should move toward integrated frameworks coupling chemistry, mechanics, and environmental durability under standardized protocols to ensure reproducibility and relevance for real applications.

Numerous studies have investigated tensile and shear strength enhancement from different angles. Studies [101,102,103] emphasize temperature effects, bond-line geometry, and surface engineering. For instance, ref. [101] ranks adhesives by shear strength at elevated temperatures, identifying thermosets as superior due to thermal stability. However, only a narrow range of thermosets was tested, leaving the performance of toughened epoxies and hybrids under thermal cycling largely unaddressed. Similarly, ref. [102] shows that ~30 μm bond-line thickness and higher loading rates improve tensile strength via strain-rate hardening, but it also reveals sharp strength declines as glass transition temperatures are approached. This aligns with polymer mechanics theory but leaves a gap: the interplay between bond-line optimization and environmental factors (humidity, UV) remains rarely examined. In [103], concrete–concrete interfaces quantified by fractal dimensions confirmed epoxy superiority in tensile and shear strength. Still, comparative data for acrylics and polyurethanes in large-scale structural systems are scarce, limiting generalization.

Bond-line geometry further illustrates trade-offs: thin lines improve load transfer but increase stress concentration, while thick lines raise toughness but reduce stiffness. Most studies, however, are confined to single-lap or double-lap geometries, limiting transferability to industrial assemblies. Bridging this scale gap remains an open challenge.

Material innovation and hybridization strategies [99,100,104,105,106] highlight how additives and fillers tailor strength. Ref. [104] reported a 54% rise in lap shear and a 240% increase in toughness with SiO_2_-based ionic nanomaterials, due to multifunctional hydrogen-bonding and ionic reinforcement. Yet, industrial feasibility and cost are rarely addressed. Similarly, ref. [100] showed optimal epoxy/alumina nanocomposite performance at 1.0–1.5 wt.% filler, but higher loading caused embrittlement, thereby contradicting other reports and stressing the need for processing–property guidelines.

Meanwhile, refs. [98,105] emphasize testing configuration. Ref. [103] used a custom compression–shear fixture, showing that hydrostatic stresses enhance joint strength, offering crashworthiness insights. Yet, such setups lack ASTM/ISO standardization, limiting uptake; broader adoption of mixed-mode protocols is needed. Study [98] explored adhesive bonding in 3D-printed PLA, finding that higher loading rates slightly improved strength but failure still initiated at the interface, highlighting weak interfacial design. Additive manufacturing–adhesive interactions remain poorly studied, particularly long-term durability. The study [99] introduced thermally conductive polyurethane adhesives with tensile strength of 10.25 MPa and shear strength of ~7 MPa, demonstrating multifunctionality for electronics and automotive use. However, improved conductivity may alter viscoelasticity, raising fatigue and creep concerns—rarely quantified in current studies. A multi-property optimization approach balancing strength, durability, and function is recommended.

**Figure 7:**
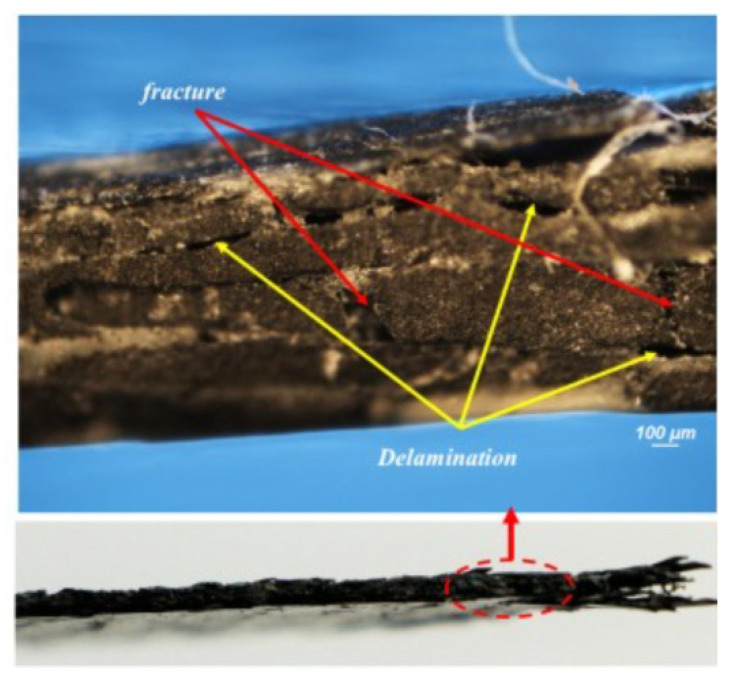
The side view of the magnified micrograph shows the deformed carbon/PPS specimen subjected to tensile loading in the 45° direction. Reprinted from Ref [107] with permission from Elsevier, 2020.

Jebri et al. [107] analyzed carbon/PPS composites under tensile loading, revealing delamination initiated by translaminar crack networks (Figure 7). While not adhesive-focused, such mechanistic studies inspire adhesive fracture research, where crack initiation mapping remains underdeveloped. Incorporating SEM/DIC evidence would improve mechanistic understanding beyond macroscopic joint tests.

**Figure 8:**
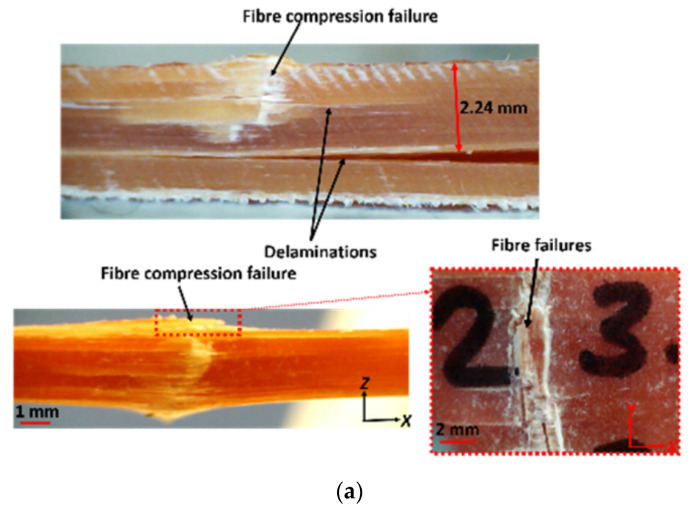

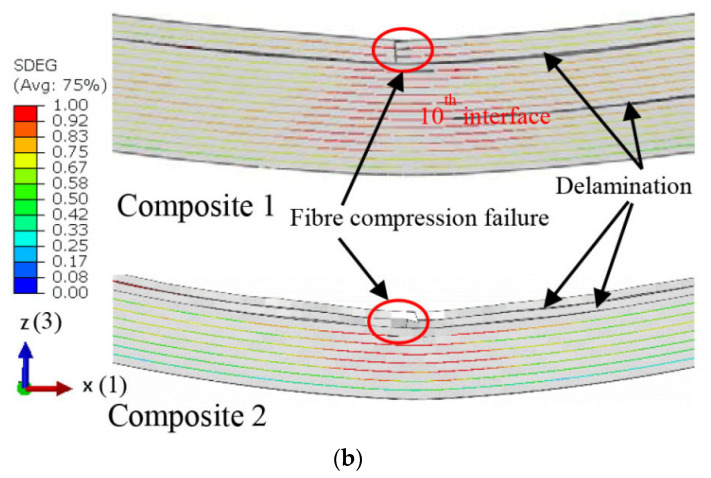
Post-deformation microscopic images of the thicker (**top**) and thinner (**bottom**) composite beams (**a**), along with the numerically derived schematics illustrating fiber failures and delamination (**b**). Reprinted from Ref [108] with permission from Springer, 2020.

**Figure 9:**
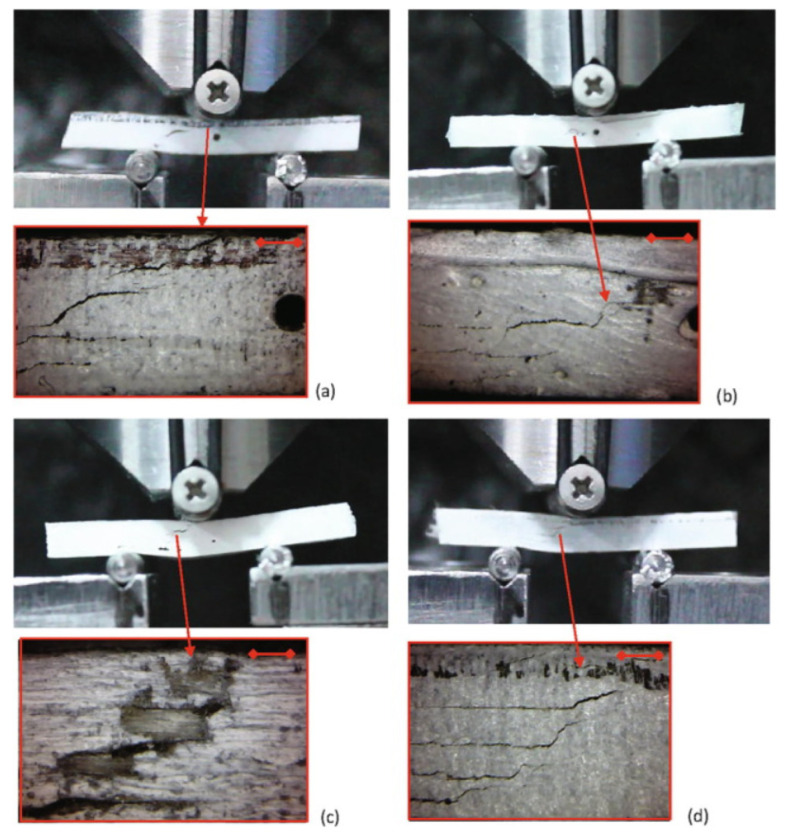
Microstructure of the fractured surfaces for the vascularized samples: (**a**) [90/0] and (**b**) [0/90]; and for the non-vascularized samples: (**c**) [90/0] and (**d**) [0/90] (Scale bar: 1 mm). Reprinted from Ref [111] with permission from Elsevier, 2020.

Demiral et al. [108] studied size effects in unidirectional GFRP bending [109,110], showing that ply number governs failure shifts from delamination to fiber compression (Figure 8). Thicker laminates exhibited flexural failure in the compression region, accompanied by delamination in two distinct zones (Figure 8a), whereas 10-ply specimens showed no visible delamination but instead failed by fiber compression (Figure 8b). A related study [111] on vascular beams showed shear-driven delamination and matrix cracking, exposing gaps in predictive modeling of complex interactions (Figure 9). Li et al. [112] optimized corrugated CorTube structures via arc sections, improving bonding but still dominated by crushing failures. This conflict illustrates that local bonding improvements do not always translate to global stability gains.

For bonded joints, single-lap, thick-adherend, and double-lap shear tests are widely employed to evaluate strength under different loading regimes [113]. Further bonded-joint work demonstrates geometry and adherend design as decisive factors. SLJs with different layups bonded with AF163-2K [114] showed failure locus shifting from adhesive to adherend with increasing off-axis plies. Increasing adherend thickness improved energy absorption by 80%, outweighing overlap length effects [115], challenging conventional design assumptions. Centelles et al. [116] investigated laminated glass under aging, revealing uneven interfacial stress, but cyclic degradation mechanisms remain insufficiently studied.

Collectively, these studies show that adhesive performance is shaped not only by chemistry but also by interfacial design, geometry, and environmental durability. Yet, predictive models for mixed-mode failures, integration with structural stability, and long-term degradation remain incomplete. Addressing these requires combining experiments with multiscale simulations and standardized durability testing.

### 3.2. Fracture Toughness and Crack Propagation

Fracture toughness is a fundamental parameter governing the failure resistance of polymer adhesives, and its reported values vary considerably across adhesive families (Table 4). A precise understanding of fracture behavior is crucial, as toughness not only dictates short-term crack initiation and growth but also determines long-term durability under cyclic loading, hygrothermal exposure, and multiaxial stresses typical of service conditions.

Epoxy systems are the most widely investigated adhesives and remain the benchmark for structural bonding in aerospace, automotive, and civil engineering. Their Mode I fracture toughness (GIc) typically lies in the 200–800 J/m^2^ range, though rubber- or nanoparticle-toughened formulations often extend to 1000–1500 J/m^2^, with rare cases exceeding 2000 J/m^2^. Mode II values (GIIc) are generally higher, around 1000–2500 J/m^2^, due to more pronounced shear plasticity. These values make epoxies highly reliable in applications demanding stiffness and load transfer. However, their performance is strongly environment-dependent: under moisture or cryogenic exposure, epoxy’s intrinsic brittleness becomes problematic, with fracture energies often decreasing by more than 40%. Various toughening strategies—including core–shell rubber particles, thermoplastic interleaves, and silica nanoparticles—have been developed to counteract these weaknesses [6,117]. Despite these advances, the literature remains inconsistent: while some studies report significant gains in toughness, others show negligible improvements or even strength reductions due to filler agglomeration or reduced crosslink density. This lack of consensus highlights the delicate balance between toughness enhancement and retention of stiffness and processability.

Polyurethane adhesives, by contrast, are characterized by their segmented molecular architecture, consisting of alternating soft and hard domains. This structure provides inherent toughness through energy dissipation mechanisms such as domain separation and large-scale plastic deformation. Reported GIc values fall between 300 and 1200 J/m^2^, and GIIc values often extend up to 3000 J/m^2^, making them considerably tougher than most epoxies. Polyurethanes also maintain toughness across a broader temperature range and show improved resistance to dynamic fatigue. Their limitations, however, stem from relatively low stiffness and diminished high-temperature performance. These deficiencies restrict their application in aerospace and defense, where dimensional stability is critical. Furthermore, their long-term resistance to UV and chemical exposure is variable, raising durability concerns. Despite these challenges, their use in automotive, construction, and renewable energy (e.g., wind blade bonding) remains widespread [118].

Acrylic adhesives, including methyl methacrylate (MMA) and cyanoacrylates, occupy an intermediate position. Their GIc values typically range from 200 to 600 J/m^2^ and GIIc from 800 to 2000 J/m^2^. Their rapid cure, tolerance to poor surface preparation, and ability to bond dissimilar substrates make them highly attractive for field applications. Yet, in unmodified form, they are relatively brittle. Rubber-toughened and nanofiller-modified acrylics can achieve toughness values comparable to lower-grade epoxies, but concerns about long-term stability remain. For example, several studies have shown that MMA adhesives exhibit stable toughness in dry laboratory conditions but degrade rapidly under cyclic humidity or immersion. Contradictory reports exist on whether environmental degradation primarily reduces GIc or shifts failure modes from cohesive to adhesive, underscoring the need for standardized protocols [119].

Thermoplastic-based adhesives such as PEEK and nylon introduce another dimension of toughness behavior. PEEK adhesives generally display GIc values of 400–1200 J/m^2^ and GIIc values of 1200–2500 J/m^2^. Their semi-crystalline microstructure provides excellent retention of toughness even at elevated temperatures, making them attractive for aerospace, oil and gas, and electronic packaging. However, their use is constrained by high cost, processing complexity, and limited commercial availability of adhesive-grade PEEK films or resins. Nylon-based adhesives (polyamide systems), particularly PA66, show GIc values of 300–900 J/m^2^ and GIIc values of 1000–2200 J/m^2^. These materials combine ductility with relatively high toughness, but their hydrophilic nature poses critical challenges. Moisture absorption leads to plasticization, lowering both stiffness and fracture toughness over time. Some studies report up to 50% reductions in fracture energy after long-term humidity conditioning. These findings have sparked debate: while nylon adhesives perform well in dry laboratory testing, their real-world service performance often proves less predictable [120].

A critical observation across all adhesive families is that fracture toughness values are not absolute material constants. They are highly sensitive to testing configuration, adhesive thickness, substrate stiffness, crack initiation method, and environmental conditioning. For example, some toughened epoxies exhibit GIc values above 2000 J/m^2^ under Mode I tests in dry conditions, yet display premature cohesive failure when subjected to mixed-mode or cryogenic loading. Similarly, while Mode II toughness is generally higher than Mode I, exceptions exist in geometries where interfacial debonding dominates, reversing the expected relationship. These inconsistencies highlight the need for standardized testing procedures and the careful reporting of experimental conditions.

**Figure 10:**
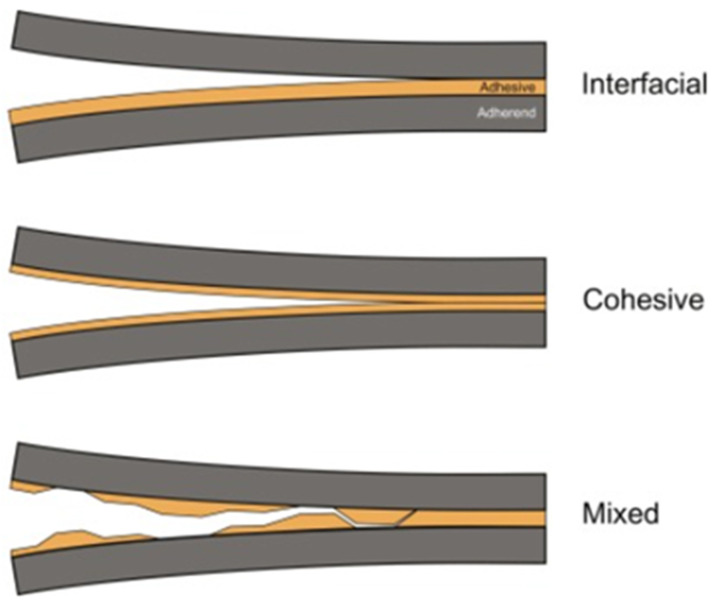
Diagrammatic illustration of adhesive joint failure modes: interfacial failure (**top**), cohesive failure (**middle**), and mixed-mode failure (**bottom**). Reprinted from Ref [126] with permission from Woodhead Publishing, 2023.

Failure mechanisms provide further complexity. Cohesive failure occurs when cracks propagate through the adhesive bulk, leaving adhesive material on both adherend surfaces (Figure 10). Microscopically, this is identified by hackle markings, river patterns, and void coalescence, often observed in epoxies and polyurethanes. SEM analysis confirms that plastic deformation zones, crazing, and shear banding frequently accompany cohesive fracture. Adhesive failure, by contrast, occurs at the interface between adhesive and substrate, producing smooth fracture surfaces and often leaving imprints of the adherend’s surface (Figure 10). Such failures are commonly associated with poor surface preparation, weak oxide layers, or moisture-induced interfacial degradation, as documented in aerospace joints. Mixed-mode failure, combining both cohesive and adhesive elements (Figure 10), is the most frequently encountered under service conditions. In wind turbine blade joints, for instance, cyclic loading often initiates cracks in the adhesive bulk before deflecting toward the interface, creating a complex hybrid failure path [121,122,123].

Surface treatment and environmental conditions strongly influence the dominant failure mode. Effective treatments, such as grit blasting, chemical priming, or plasma activation, raise interfacial strength above that of the adhesive bulk, shifting failure from adhesive to cohesive or mixed. A recent study demonstrated that plasma-treated CF/PEKK bonds achieved a 238% increase in lap-shear strength and transitioned from adhesive to cohesive/mixed failures [124]. Conversely, environmental degradation frequently reverses this trend, promoting adhesive failures. For example, CFRP/titanium joints subjected to hygrothermal aging at 40 °C and 95% RH initially failed cohesively but shifted to adhesive failures after 240 h of exposure. These results underline the significant role of hygrothermal effects in long-term durability [125].

Fracture toughness is a key parameter describing an adhesive’s resistance to crack initiation and growth, typically measured through standardized tests. Mode I (opening) is characterized by the Double Cantilever Beam (DCB) test, Mode II (shear) by the End-Notched Flexure (ENF) test, and mixed-mode conditions by the Mixed-Mode Bending (MMB) test [127]. Crack propagation pathways differ between composites and bonded joints: in laminates, fiber bridging can delay crack growth and increase toughness, whereas in adhesive joints, cracks often propagate along the bondline, with interfacial debonding occurring if adhesion is weak [128].

**Figure 11:**
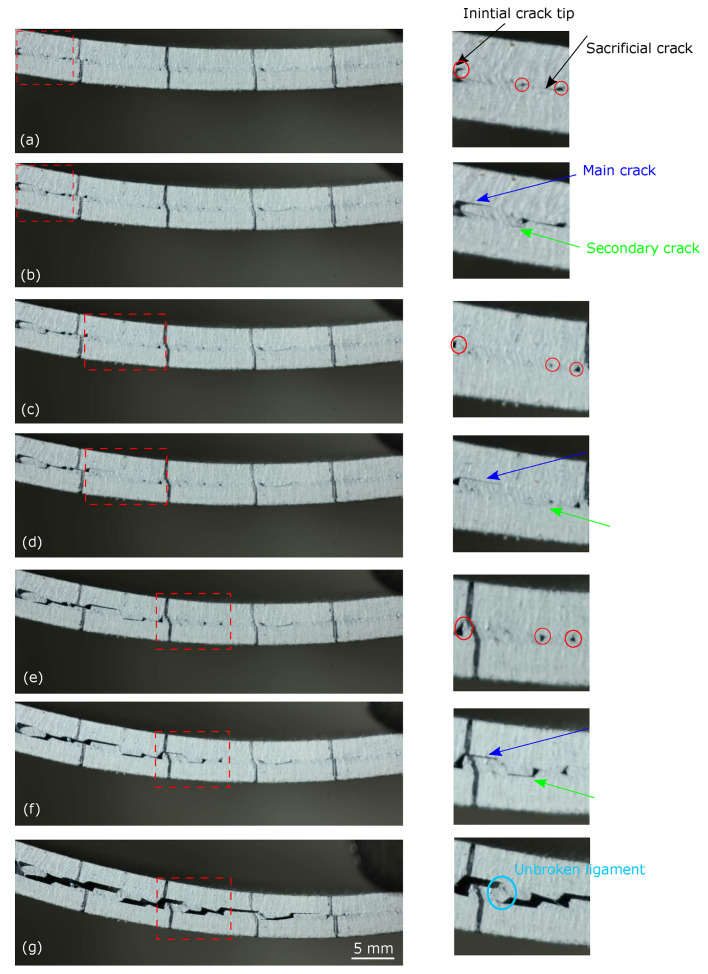
An example illustrating the sequence of crack propagation (from (**a**–**g**)) during the ENF test on a modified adhesive featuring a sacrificial crack. Reprinted from Ref [129] with permission from Elsevier, 2021.

To improve toughness, ref. [129] proposed introducing sacrificial cracks using polytetrafluoroethylene (PTFE) films (Figure 11). This approach redistributed strain in ENF tests with CFRP adherends, delaying crack propagation and raising initiation toughness, propagation toughness (GIIc), and maximum load capacity (Pmax). Gains of 96% in initiation toughness, 98% in GIIc, and 25% in Pmax were reported for a 2 mm crack width with a 5 mm gap. While effective at both thin and thick bondlines, the method has mainly been validated at the coupon scale, and its transferability to large or complex joints under multiaxial stresses remains uncertain.

Beyond sacrificial cracking, bio-inspired and structural strategies are increasingly explored. Wagih et al. [130] mimicked natural toughening mechanisms, embedding sacrificial cracks to promote crack bifurcation and ligament bridging, thereby raising interfacial toughness. Study [131] toughened epoxy by adding thermoplastic polyamide inclusions, shifting failure from brittle to ductile and enabling stable crack growth through progressive energy dissipation. Delzendehrooy et al. [132] advanced prediction methods, training an artificial neural network (ANN) on literature data to identify key parameters (adherend modulus, crack length, loading rate). ANN models successfully ranked influential factors but face limitations: they rely heavily on existing datasets and may not generalize to emerging systems such as bio-based or nanomodified adhesives. This gap underscores the need for large, integrated databases that combine experimental, numerical, and molecular-scale insights.

Bondline thickness and durability also play decisive roles. Study [133] showed that increasing thickness enhances fracture energy but can induce nonlinear effects and stick–slip crack propagation at high loading rates. Similarly, ref. [134] reported that thicker bondlines improved fatigue toughness under cyclic loading, though with a trade-off in higher initial compliance. Bio-based alternatives are attracting attention: Saleh et al. [135] demonstrated that glycidyl phosphate ester formulations doubled Mode I toughness in CFRP joints and shifted failures from adhesive to cohesive/mixed modes. While promising, the long-term durability and processing challenges of such bio-based systems remain underexplored, representing an important research gap.

Environmental durability adds another layer of complexity. Houjou et al. [136] investigated moisture-induced degradation of epoxy joints in DCB tests. Moisture exposure altered fracture mode from cohesive to adhesive and back again, depending on conditioning time, while surface treatments such as sandblasting partially preserved cohesive behavior. Strikingly, even when GIC values increased, cyclic crack growth rates (da/dN) did not consistently decrease, highlighting the disconnect between quasi-static fracture toughness and long-term fatigue resistance. This unresolved contradiction points to a broader challenge: short-term toughness metrics do not fully capture service-relevant durability.

Various toughening approaches have been trialed to mitigate these limitations. Electrospun nylon-6 nanofiber interleaves in carbon/epoxy laminates improved total toughness by ~27%, post-peak plastic toughness by ~46%, and the load for matrix cracking by ~65%, provided the areal weight was optimized [137]. Rubber toughening has also been extensively studied: liquid rubber and thermoplastic particles raised fracture toughness from ~9.5 kJ/m^2^ to ~32.6 kJ/m^2^ (243% increase) compared to neat epoxy, although at the expense of higher viscosity and lower crosslink density [138]. Veil interleaving (e.g., TPPI fiber veils in CF/TSPI composites) achieved even greater gains (~179% in Mode I and ~132% in Mode II toughness) without compromising thermal stability when areal density was tuned.

However, these approaches are not without drawbacks. Higher filler or interleaf contents often increase resin viscosity, reduce flow, and lead to voids or incomplete wetting. Some interleaves can reduce in-plane stiffness or strength, while cost and weight penalties may offset mechanical benefits. Moreover, when the adhesive matrix already possesses high toughness, further improvements from interleaves or nanofillers are often lower than theoretical predictions.

Collectively, these studies reveal that enhancing fracture toughness and controlling crack propagation in adhesive joints is a multifactorial challenge shaped by adhesive chemistry, joint architecture, loading rate, bondline thickness, and environmental conditions. Bio-inspired crack bifurcation, bio-based formulations, and advanced modeling frameworks offer promising directions, yet major research gaps persist in linking laboratory-scale metrics to real-world fatigue and durability performance. Bridging these gaps will require integrating mechanical testing, environmental conditioning, and multi-scale modeling into unified design frameworks capable of predicting adhesive performance in service.

### 3.3. Fatigue and Long-Term Durability

Polymer adhesives in composite structures and bonded joints are routinely subjected to cyclic loading during service, leading to progressive fatigue damage over time. The fatigue performance of these adhesives is therefore a decisive factor in ensuring the long-term durability and safety of bonded structures. Fatigue behavior is generally characterized through cyclic loading tests, where fatigue crack growth rates and stress–life (S–N) curves provide essential insights into endurance under repeated stresses. Such tests capture the progressive deterioration of adhesive joints and composite matrices under fluctuating loading conditions, enabling a more realistic assessment of service performance compared to quasi-static evaluation [90,139,140].

Despite its importance, the fatigue life of adhesive systems remains far less systematically reported than their static strength or fracture properties. Table 5 summarizes typical fatigue life ranges reported for different classes of adhesives, including epoxies, polyurethanes, acrylics, PEEK-based systems, and nylon-derived adhesives under cyclic loading. Epoxy adhesives typically achieve fatigue lives of 10^5^–10^7^ cycles, particularly in high-strength structural joints. Their combination of stiffness and strong adhesion to metals and composites renders them reliable for long-term structural applications. However, epoxy systems display high sensitivity to environmental conditions such as humidity and temperature fluctuations, which accelerate crack initiation and propagation, thereby undermining fatigue resistance. Furthermore, substantial scatter is reported depending on surface preparation and curing protocols, making it difficult to generalize epoxy fatigue life across different formulations.

Polyurethane adhesives generally exhibit fatigue lives in the 10^4^–10^6^ cycle range (Table 5). Their inherent elasticity and energy dissipation make them well-suited for applications demanding vibration damping and flexibility, such as in automotive and construction joints. At the same time, their relatively lower stiffness compared with epoxies can cause earlier fatigue failure under high static loads. Another drawback is their vulnerability to moisture and chemical environments, which tends to degrade performance more rapidly than in epoxy-based systems. Consequently, polyurethane fatigue performance is highly dependent on specific formulations and service conditions.

Acrylic adhesives, by contrast, usually show fatigue lives between 10^4^ and 10^5^ cycles (Table 5), lower than both epoxies and polyurethanes. While their rapid curing and strong initial bond strength make them attractive in mass-production contexts, their relatively poor resistance to cyclic loading and environmental aging limits their suitability for long-term structural use. In particular, ultraviolet and humidity exposure accelerate fatigue crack growth, which helps explain their restricted application in critical load-bearing or fatigue-prone environments.

PEEK-based adhesives demonstrate outstanding fatigue lives, reaching 10^6^–10^8^ cycles (Table 5). Their semicrystalline nature, toughness, high glass-transition temperature, and chemical stability make them highly promising for aerospace and energy-sector joints exposed to cyclic stresses and aggressive environments. Nevertheless, widespread adoption is hindered by high costs and processing challenges. Moreover, available fatigue data for PEEK adhesives remain sparse compared to epoxies, and variability is often linked to reinforcement types or manufacturing methods. For instance, bulk and 3D-printed PEEK structures show distinct fatigue responses. At stress amplitudes below ~75% of ultimate tensile strength, many specimens achieve “run-out” behavior (survival beyond 10^6^–10^7^ cycles), suggesting a fatigue endurance limit. Conversely, at stress amplitudes approaching 92–95% of tensile strength, fatigue life rapidly falls to 10^4^–10^5^ cycles. This contrast underscores the duality of PEEK: while intrinsically tough, it is highly sensitive to microstructural defects—particularly in additively manufactured forms—making service-life prediction challenging.

For nylon-based adhesives, systematic fatigue-life data are notably lacking. Most available research addresses polyamide substrates or polymer blends rather than dedicated nylon adhesive formulations and bonded-joint fatigue. As such, no consistent fatigue ranges are provided for nylon adhesives in Table 5. This absence constitutes a significant gap in the literature and highlights an urgent need for targeted studies, especially given the wide use of nylon-based systems in automotive, consumer, and electronic applications.

**Table 5:** The typical fatigue life of an adhesive system.

Adhesive System	Fatigue Life Range/Key Data	Key Notes and Reference(s)
Epoxy	~10^5^–10^7^ cycles under favorable shear/tensile fatigue; significantly reduced life when moisture or environmental degradation is present	Data from a cold-curing structural epoxy investigated under wet/dry fatigue: up to ~10 million cycles in benign conditions, with moisture dramatically reducing fatigue life [141,142,143,144].
Polyurethane (PU)	~10^4^–10^6^ cycles in normalized cyclic shear/tension conditions; fatigue endurance depends strongly on temperature and environmental conditions	Fatigue design curves and S–N behavior for a one-component PU adhesive show wide cycle ranges and environmental sensitivity [145,146].
Acrylics (MMA/structural acrylics)	~10^4^–10^5^ cycles in structural adhesive fatigue tests; some data suggest improved endurance for modified formulations	Plexus MA300 methacrylic adhesive fatigue data provide S–N results for a structural acrylic system [147].
PEEK-based systems	~10^6^–10^8^ cycles. Few direct fatigue data available for adhesive joints; bulk PEEK fatigue studies show endurance, but joint-level fatigue data are scarce	Existing PEEK fatigue studies focus on bulk polymer or composite fatigue, not specifically adhesive joints, so fatigue life in bonding scenarios remains uncertain [148].
Nylon-based adhesives	Very limited fatigue life information for nylon adhesives themselves; fatigue performance is mostly derived from joint-level studies involving polyamide substrates	Systematic data for commercial nylon adhesives under cyclic loading remains scarce.

Across application domains, studies [149,150,151,152,153,154,155] reinforce the centrality of fatigue resistance in ensuring long-term adhesive durability. Several recurring themes emerge: interfacial degradation, environmental exposure, and dynamic stress all play critical roles in governing service life. For instance, ref. [149] demonstrated that fatigue testing provided far more sensitive discrimination of bonding protocols in composite repairs than quasi-static methods, revealing that no adhesive fully restored the cohesive strength of aged composites. However, incorporating a silane coupling agent significantly improved endurance, emphasizing the role of interface chemistry. In dental systems, ref. [151] showed that resin–dentin bond fatigue is highly dependent on etching protocols and adhesive application modes, suggesting that fatigue tests are more representative of clinical durability than static tests. Complementing these observations, ref. [153] reported that adding microcrystalline cellulose (MCC) to moisture-curable polyurethane enhanced both fatigue resistance and hygrothermal stability, outperforming sawdust-filled analogs. Collectively, these findings highlight the gap between laboratory fatigue testing and real service conditions, particularly where combined moisture and thermal cycling accelerate degradation.

From a civil infrastructure perspective, ref. [150] demonstrated that toughened epoxy adhesives in CFRP–steel joints extended steel bridge service life by up to 7.9 times under fatigue, illustrating the potential of optimized formulations for dynamic load environments. Similarly, ref. [154] compared FRP–concrete joints and showed that rubber-modified epoxies outperformed stiffer formulations in fatigue endurance and damage tolerance. These results highlight a critical trade-off: while stiffness improves initial static capacity, increased compliance can extend fatigue resistance, suggesting system-specific optimization.

In biomedical applications, ref. [152] developed a dual-network hydrogel that preserved anti-fatigue properties under repeated loading, while [155] proposed a topologically cross-linked hydrogel capable of sustaining wet-surface adhesion over extended durations (>125 kPa adhesion strength for 70 days), enabling stable electrophysiological signal monitoring in vivo. These studies underline the role of cross-linking architectures in controlling fatigue and durability in biological environments, though scalability and long-term biocompatibility remain unresolved.

**Figure 12:**
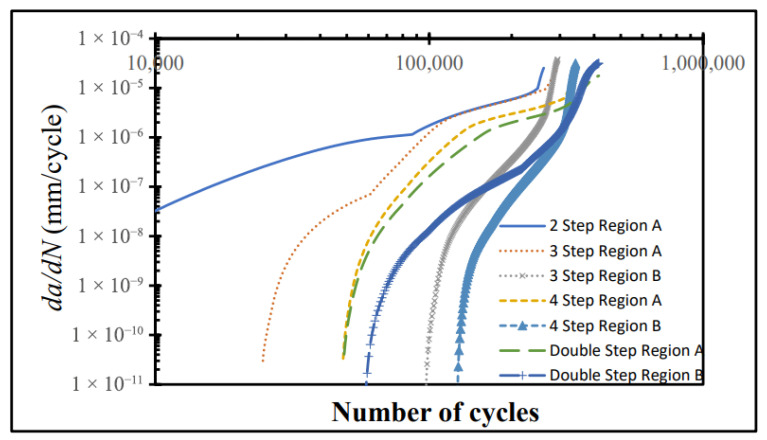
The variation in crack growth rate (*da*/*dN*) with respect to the number of loading cycles was analyzed at different regions within the adhesive layer of lap joints featuring various step configurations under cyclic tensile loading [158].

Cyclic stresses typically initiate microscopic cracks at adhesive–substrate interfaces or internal flaws, which eventually coalesce into catastrophic failure [156]. Interestingly, Demiral et al. [157] observed that in SLJs under tensile fatigue, higher applied loads delayed crack initiation, with cracks instead appearing near final failure—a counterintuitive result suggesting complex load–damage dynamics. Separately, ref. [158] showed that stepped-lap joints with a double-step geometry exhibited 21.8% longer fatigue life than four-step joints (Figure 12), highlighting the critical influence of joint geometry on durability. However, the absence of consensus on standard joint geometries for fatigue testing continues to hinder cross-comparison and data consolidation across studies.

In conclusion, the fatigue and long-term durability of adhesives depend on multiple interdependent factors: initial bond strength, adhesive formulation, interfacial chemistry, joint design, and environmental stability. Recent advances—such as nanofiller incorporation, cellulose-derived additives, and cross-linked hydrogels—demonstrate clear improvements in fatigue resistance. Yet, persistent gaps remain: translating coupon-level data to full-scale structural predictions, standardizing fatigue testing protocols across industries, and linking short-term endurance limits to real-world service lifetimes. Addressing these challenges will be critical for enabling adhesives to meet the stringent durability requirements of aerospace, civil engineering, biomedical, and electronic applications.

### 3.4. Creep and Viscoelastic Behavior

The viscoelastic nature of polymer adhesives means that their mechanical properties evolve over time under sustained or cyclic loading. Two time-dependent processes—creep and stress relaxation—are central to understanding long-term performance. Creep refers to gradual elongation or displacement under constant stress, while relaxation describes the reduction in stress under constant strain. Both phenomena redistribute loads in bonded assemblies: relaxation can reduce peak stresses at stress concentrators, while creep may progressively weaken joints and compromise stiffness. For structural applications, durability is therefore governed by a coupled assessment of creep (constant-load behavior) and relaxation (constant-strain response) [159,160].

Creep resistance is typically evaluated using long-term static loading tests and accelerated methods, such as elevated temperature and humidity exposure, often combined with time–temperature superposition to extrapolate lifetime behavior. For pressure-sensitive adhesives (PSAs) and soft adhesives, recent studies reveal complex multi-phase creep (primary, secondary, tertiary) that cannot be captured by single-term linear models. This has motivated the adoption of generalized Maxwell, Burgers, or multi-element viscoelastic formulations to describe their full time-dependent response [161]. Dynamic mechanical analysis (DMA) has also become essential for probing adhesive transitions between elastic and viscous states, enabling the prediction of performance under service-like conditions. Temperature, humidity, and applied stress levels are primary variables influencing creep. Elevated temperatures accelerate molecular mobility within the adhesive matrix, promoting deformation and reducing service life, while humidity can induce plasticization, lowering stiffness and exacerbating creep. Formulation strategies such as increased crosslink density and the incorporation of nanofillers have therefore been explored to suppress molecular mobility, enhance stability, and improve creep resistance [162,163].

Methodological advances have also expanded characterization approaches. Dedicated cyclic creep rigs for SLJs, especially in PSA systems, and combined experimental–numerical frameworks now allow calibration of viscoelastic models using creep–recovery and relaxation data. These methods enable prediction of joint-level performance under realistic service loading, while also confirming that creep cannot be inferred from bulk adhesive behavior alone. Substrate stiffness, bondline thickness, and interfacial adhesion strongly influence joint creep and must be included in predictive frameworks [164].

**Figure 13:**
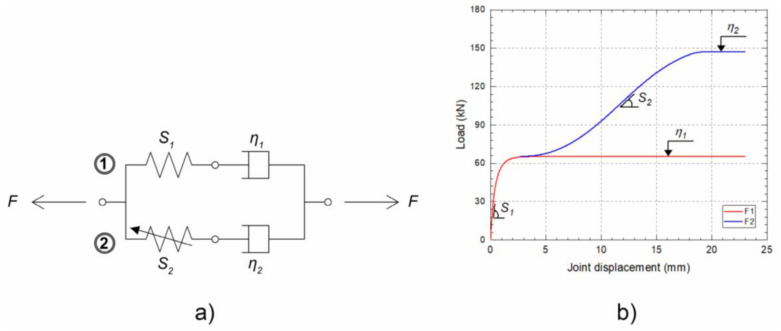
Rheological structure of phenomenological model: Two dissimilar parallel Maxwell units (**a**) and the corresponding load displacement curves (**b**). Reprinted from Ref [165] with permission from Elsevier, 2022.

At the modeling level, Eslami et al. [165] introduced a phenomenological framework (Figure 13) that uses two parallel Maxwell elements—one linear and one nonlinear—to reproduce the rate-dependent load–displacement response of pseudo-ductile joints under monotonic and cyclic loading. Calibrated against experimental data, the model captured strain hardening, softening, and rate effects through power-law relations linking viscoelastic parameters to displacement rates and cycle counts. While effective, this approach remains system-specific, highlighting the need for broader validation across adhesive chemistries and joint geometries. Similarly, ref. [166] emphasized the critical role of rheology in PSAs, showing how flow during bonding and debonding governs shear, tack, and peel performance. Despite their widespread industrial importance, PSAs remain underexplored in predictive modeling of long-term viscoelastic reliability.

A series of studies [60,96,167,168,169,170,171] collectively emphasize the complex time-dependent responses of adhesives, particularly under combined environmental and mechanical stress. A consistent theme in [96,167,168] is the integration of viscoelastic modeling with curing and environmental effects. For instance, ref. [167] developed an FE model capturing nonlinear viscoelastic–viscoplastic behavior of scarf joints, showing that viscoplasticity redistributed stresses and reduced local concentrations—an encouraging insight for joint design. In [168], hygrothermal aging reduced modulus and creep resistance in epoxies, but predictions using a generalized Kelvin model provided good accuracy. Similarly, ref. [96] demonstrated that higher curing temperatures improved creep resistance, again well described by Kelvin-type models. These results confirm the utility of classical viscoelastic models but also expose their limitations: they often require case-by-case recalibration, restricting transferability across adhesives and service environments.

To mitigate time-dependent degradation, adhesives are frequently modified through enhanced crosslinking, nanofiller addition, or graded interfaces. These strategies lower creep compliance and improve load transfer but often introduce trade-offs with toughness, processability, or curing complexity. This underscores a broader research gap in multi-parameter optimization, where both experimental characterization and hybrid viscoelastic–damage models are required to balance competing requirements for aerospace, automotive, and civil engineering systems [160].

Formulation–microstructure relationships further shape viscoelastic performance. In [169], polyurethane adhesives for cross-laminated timber were studied using creep tests combined with Maxwell modeling and FE simulation, which accurately reproduced structural deflections. In [170], a unified creep model for PSAs successfully captured all three creep phases under varying stress and temperature, providing a rare framework for long-term prediction. Conversely, ref. [171] highlighted that bulk properties are poor predictors of joint-level creep in hot-melt PSAs, particularly for low-Tg formulations. This conflict underscores the critical role of interfacial and geometric factors in defining performance, a recurring limitation in adhesive durability prediction. In the biomedical field, ref. [60] examined resin–dentin adhesives, where both static and dynamic creep altered bond strength and failure modes. FE analysis confirmed that creep redistributed interfacial stresses, improving durability—a result that contrasts with the generally negative view of creep in structural contexts.

Together, these findings illustrate that while viscoelastic modeling can capture many key mechanisms, predictive reliability across different adhesives, environments, and geometries remains inconsistent. Classical models often oversimplify adhesive–substrate interactions and environmental effects, while advanced multi-element or phenomenological models are not yet broadly transferable. Persistent gaps therefore remain in three areas: (i) developing transferable viscoelastic–damage models that account for both bulk and interfacial effects, (ii) standardizing creep and relaxation protocols across industrial and biomedical sectors, and (iii) linking bulk adhesive properties to realistic joint-level behavior, particularly in PSAs and bio-adhesives where variability and environmental sensitivity are greatest.

In summary, creep and viscoelasticity represent critical, but incompletely resolved, challenges in predicting the long-term durability of polymer adhesives. Advances in testing, modeling, and formulation have provided valuable insights into time-dependent deformation mechanisms, yet systematic frameworks that unify environmental effects, interfacial behavior, and joint-scale mechanics remain elusive. Addressing these challenges will be central to ensuring adhesive reliability across aerospace, automotive, civil infrastructure, and biomedical applications.

### 3.5. Impact and High-Strain-Rate Behavior

Polymer adhesives in composite structures and bonded joints must efficiently absorb and dissipate impact energy to ensure structural integrity and mitigate catastrophic failure. Their resistance under impact is governed by adhesive toughness, bond-line thickness, and substrate compatibility, with the balance among these parameters dictating overall joint reliability. Epoxies remain the dominant choice in high-performance systems due to their high strength and stiffness; however, their intrinsic brittleness necessitates the use of toughened variants. Modifications with elastomeric or thermoplastic phases improve crack resistance and enhance energy dissipation, thereby delaying crack initiation and growth [172,173]. Beyond traditional toughening, hybrid formulations incorporating nanoreinforcements such as graphene, silica, and carbon nanotubes (CNTs) provide additional mechanisms of crack deflection and bridging, which strengthen resistance to impact and fracture [174]. Nevertheless, challenges related to reproducibility, processing scalability, and the reliable integration of nanomaterials continue to hinder industrial adoption, highlighting the gap between laboratory innovations and manufacturable solutions.

Another defining characteristic of adhesives is their strong strain-rate sensitivity. At low strain rates, many adhesives display ductile behavior, accommodating deformation by energy dissipation. However, under high strain-rate conditions, rapid stress accumulation often triggers a brittle response, resulting in sudden fracture [175]. Characterization of such behavior relies heavily on dynamic mechanical analysis (DMA) and Split Hopkinson Pressure Bar (SHPB) testing [176]. Recent studies with nanofillers report enhanced toughness and energy absorption at elevated strain rates [177]. Yet, a persistent research gap lies in translating nanoscale toughening mechanisms into reliable macroscale design principles, particularly for full joint geometries under service-relevant conditions such as automotive crashes or blast loads. Bridging this scale gap remains a pressing need for both academic and industrial communities.

**Figure 14:**
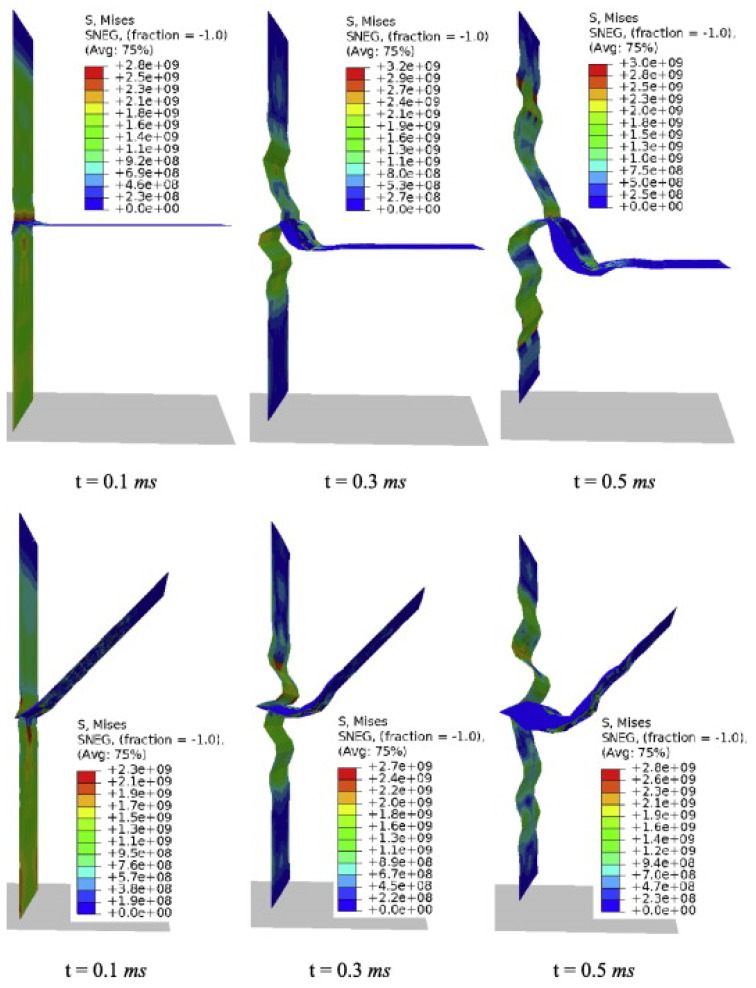
Impact progression at various time intervals for different joint configurations: the upper sequence corresponds to the 0° joint, while the lower sequence represents the 45° joint. Reprinted from Ref [180] with permission from Elsevier, 2015.

In practical applications, strain-rate-dependent adhesive behavior is particularly critical. In the automotive sector, crash-resistant adhesives enhance energy absorption and crashworthiness, while also enabling lightweight design strategies [178]. Similarly, in aerospace, bonded joints play a vital role in mitigating damage under extreme impact events such as bird strikes [179]. Moreno et al. [180] demonstrated that increasing joint angles can significantly enhance crash energy absorption due to the promotion of adhesive damage, a finding with direct implications for joint design optimization (see Figure 14). Beyond transportation, adhesives are employed in construction and defense for blast-resistant systems, where they mitigate shockwave propagation and delay delamination [181]. However, there exists a persistent conflict between the demand for high toughness and the constraints imposed by curing speed, processing scalability, and cost. This tension underlines the necessity of integrated design strategies that account for both performance and manufacturability.

The mechanical response of adhesives under dynamic loading is inherently formulation-dependent and influenced by interrelated factors such as adhesive thickness, substrate stiffness, and environmental conditions. Advances in numerical tools, including finite element methods and cohesive zone models, have significantly improved the predictive capability of adhesive failure simulations [182]. Yet, current models struggle to capture multi-scale interactions—for instance, how nanoscale filler toughening interacts with joint-level fracture processes. A key research need is the development of multi-physics, multi-scale modeling frameworks that integrate atomistic, meso-, and macro-level phenomena. Such approaches, calibrated against experimental results across a wide range of strain rates and environments, could provide predictive reliability for real-world structures where safety margins are critical.

Several studies have provided important insights into the high-strain-rate behavior of adhesives [183,184,185,186,187,188,189,190]. In [183], epoxy adhesives were systematically tested under tensile loading across a broad range of strain rates, from quasi-static to extremely high speed. The results revealed that both yield stress and tensile strength increase with strain rate, implying that adhesives stiffen under rapid loading. Importantly, this study proposed a predictive method for estimating high-rate strength and creep resistance from standard tensile tests, offering a practical link between conventional testing and extreme loading conditions.

The influence of chemical composition on rate sensitivity has also been highlighted. In [186], polyurethane adhesives synthesized from different polyol and chain extender combinations were examined under varying rates and temperatures. Certain formulations showed rate- and temperature-insensitive behavior, whereas others—especially those containing PPG and PCD polyols—exhibited severe embrittlement under high strain rates combined with low temperatures. This underscores the role of chemical architecture in dictating strain-rate and temperature performance, suggesting that formulation-specific guidelines are needed for designing adhesives for impact-critical applications.

Studies [184,185,188] provide deeper insight into fracture behavior and joint-level performance. For example, ref. [184] emphasized the distinction between nominal loading rate and the actual strain rate at the crack tip during mode I fracture testing of polyurethane adhesives. The findings revealed that strain rate at the crack tip varies significantly under constant loading, especially at high test speeds and low temperatures, exposing the complex interplay between adhesive thickness, temperature, and crack propagation dynamics. In [185], mixed-mode fracture experiments revealed that fracture energies (mode I and II) increased dramatically—by up to 95 times—with increasing strain rate, particularly above the glass transition temperature (Tg). This strong coupling between temperature and strain rate highlights the necessity of accounting for thermo-mechanical interactions when designing adhesives for automotive crashworthiness. Similarly, SHPB experiments conducted in [188] on Al/PMMA joints showed that bond strength reached an optimum at a specific adhesive thickness under high strain rates. Moreover, failure modes transitioned from interfacial to mixed, as altered stress distributions favored cohesive contributions—an insight directly relevant for optimizing joint thickness in automotive structures.

The dynamic performance of other adhesive systems has also been explored. In [190], structural vinylester adhesives exhibited pronounced strain-rate sensitivity, with improvements in toughness, strength, and plastic deformation capacity under rapid loading. Remarkably, despite large deformations, no macroscopic damage was visible, and the adhesives displayed substantial energy absorption capacity as confirmed by energetic analyses. These results position vinylester adhesives as promising candidates for high-strain-rate applications in energy-absorbing bonded structures. Complementary findings in [189] demonstrated that density and expansion thickness govern the response of cellular adhesives, while [187] provided data on stiff clay bonding under impact conditions, broadening the scope of adhesives considered for extreme environments.

Collectively, these studies confirm that adhesives are strongly rate-sensitive, with mechanical properties and failure mechanisms heavily dependent on strain rate, adhesive chemistry, temperature, and joint geometry. However, several gaps and conflicts remain unresolved. A critical bottleneck is the lack of standardized testing protocols, which complicates cross-study comparisons and hinders the establishment of universal design guidelines. Moreover, while laboratory-scale insights provide valuable mechanistic understanding, their translation into industrial-scale joints and assemblies remains difficult. In particular, linking strain-rate data obtained from bulk adhesive specimens to complex bonded structures under real-world crash or blast conditions is a major challenge. Addressing these gaps requires not only advanced experimental methods and multi-scale simulations, but also closer collaboration between academia and industry to develop application-specific standards.

## 4. Applications and Challenges

### 4.1. Applications

Despite the numerous advantages of polymer adhesives in composite structures, several persistent challenges and limitations restrict their wider and more reliable application. Among these, adhesive thickness optimization, durability under harsh environments, and manufacturing-related issues such as surface preparation, curing conditions, and quality control are particularly critical.

One of the most influential parameters is adhesive thickness, which directly governs stress distribution, stiffness, and overall joint performance. A thin bond line typically enhances load transfer efficiency but increases susceptibility to stress concentrations and premature failure. Conversely, a thicker adhesive layer can improve toughness and impact resistance but may reduce shear strength due to higher compliance [191]. In composite matrices, identifying the optimal thickness is essential for uniform load distribution while minimizing voids and microcracks that act as defect initiation sites. However, achieving this balance requires precise control during manufacturing, making thickness optimization a complex engineering challenge where experimental findings often conflict across different material systems.

Another major concern is durability in harsh environmental conditions such as extreme temperatures, humidity, and chemical exposure [192]. Elevated temperatures accelerate polymer degradation, leading to reduced adhesion strength and mechanical performance. Many adhesives are prone to thermal softening or embrittlement under fluctuating thermal cycles, while moisture absorption can further degrade bonds through hydrolysis, plasticization, or interfacial weakening. Similarly, exposure to fuels, solvents, and acidic or alkaline environments compromises long-term performance. Addressing these durability concerns requires the development of adhesives with improved thermal stability, moisture resistance, and chemical inertness. Yet, a gap persists between laboratory-scale formulations with promising resistance and industrial systems that must perform consistently under multi-degradation environments.

Manufacturing challenges add further complexity. Surface preparation is critical to ensure strong adhesion, since contamination, oxidation, or insufficient roughness can lead to weak bonding [20,193]. Advanced treatments such as plasma activation, corona discharge, and chemical primers have been shown to markedly improve strength, durability, and failure modes. For instance, in carbon fiber-reinforced epoxy (CF/EP) composites, low-temperature plasma treatment increased tensile shear strength of single-lap joints by ~119.6% compared to untreated surfaces, with the failure mode shifting from interfacial to cohesive-dominated failure—a sign of stronger adhesive-substrate interaction [194]. Similarly, in adhesive-bonded CFRP joints, atmospheric-pressure plasma treatment (APPT) improved lap-shear strength from ~8.6 MPa (untreated) to ~31.6 MPa under optimized conditions, representing nearly a four-fold increase [195]. In thermoplastic composites such as CF/PEKK, plasma treatment boosted lap-shear strength from ~8.35 MPa (untreated) to ~28.28 MPa under favorable parameters (~238% improvement) [121]. Corona discharge also demonstrated significant improvements: for polypropylene bonded with PU adhesive, corona treatment enhanced adhesion due to the creation of interfacial chemical bonds [196]. Likewise, structural primers have been shown to markedly improve the durability and corrosion resistance of aerospace aluminum alloy joints compared to untreated or only cleaned surfaces [197].

However, while these treatments clearly enhance adhesion, they also introduce new engineering trade-offs. Determining optimal plasma exposure time, nozzle distance, or power settings requires careful calibration to avoid damage, over-etching, or formation of weak boundary layers—risks especially relevant for low-surface-energy or polymer-rich adherends. Moreover, such treatments increase process complexity, cost, and time, raising questions about scalability for mass production.

Beyond surface preparation, curing conditions are another decisive factor for adhesive performance. Variations in curing time, temperature, and pressure affect crosslinking density and thus the mechanical properties of the adhesive. Inadequate curing may leave residual stresses, incomplete polymerization, or weakly bonded interfaces that drastically reduce service life [27]. Outgassing during curing is also critical, as trapped volatiles or moisture can create voids and porosity, compromising structural integrity and durability. These issues are compounded in large-scale production, where maintaining consistent curing profiles across large components is inherently difficult.

Defect detection adds a further challenge, since voids, inclusions, or weak bonds may not be visible through standard inspections. Advanced non-destructive evaluation (NDE) methods such as ultrasound and X-ray computed tomography are increasingly used to assess adhesive quality, but their implementation introduces cost and logistical hurdles. This underscores a persistent gap between laboratory-scale bonding studies—often conducted under carefully controlled conditions—and industrial-scale manufacturing, where variability, defect detection, and cost-efficiency are central concerns.

In summary, although adhesives play a transformative role in enabling lightweight and damage-tolerant composite structures, their performance remains highly sensitive to thickness, environmental durability, surface treatment, and curing conditions. Current advances in plasma treatments, primers, and curing control show significant promise, but unresolved conflicts persist between performance enhancement and industrial feasibility. Future progress requires integrated approaches that address thickness optimization, develop environmentally robust adhesive formulations, and establish standardized manufacturing and inspection protocols. Without such holistic strategies, the gap between laboratory findings and reliable large-scale applications will remain a major barrier to the broader adoption of adhesive bonding in critical industries.

### 4.2. Challenges

Advancements in adhesive technology are critical for overcoming existing challenges and broadening the application of polymer adhesives in composite structures. A central research direction focuses on the development of high-performance adhesives with improved mechanical robustness and enhanced environmental resistance. The incorporation of nanomaterials such as carbon nanotubes, graphene, or silica nanoparticles has shown significant potential to enhance fracture toughness, thermal stability, and overall durability. These nanomodified adhesives exhibit improved load-bearing capacity and resistance to degradation, positioning them for demanding applications in aerospace, automotive, and structural engineering. Complementing this strategy, hybrid adhesives that combine thermosetting and thermoplastic features are also being developed, offering a balance of toughness, flexibility, and processability [198].

Another promising line of innovation involves smart adhesives with self-healing capabilities [199]. These materials can repair damage via intrinsic or extrinsic mechanisms. Intrinsic self-healing relies on reversible interactions such as dynamic covalent bonds (e.g., disulfide bonds) or non-covalent interactions like hydrogen bonding, while extrinsic approaches incorporate healing agents (microcapsules, vascular networks) that are released upon crack formation. Experimental studies illustrate the potential: Wang et al. [200] prepared an epoxy cured with a disulfide-containing agent (dimethyl 3,3′-dithiodipropionate + polyether amine), achieving ~98% healing efficiency under mild conditions (60 °C, 6 h) with exceptional elongation (~795%) and effective crack repair in coatings. Sáiz et al. [201] demonstrated that polymers with disulfide bonds could undergo repeated exchange reactions, with healing behavior strongly dependent on crosslinker type and network structure. Niu et al. [202] introduced a multifunctional epoxy with both disulfide and hydrogen bonding, enabling repeated self-healing and adhesive re-bonding. The integration of such systems into structural composites could reduce maintenance, enhance safety, and extend service life. However, important performance metrics—including healing efficiency, repeatability of healing cycles, stimulus conditions (temperature, light, pH), and retained strength after healing—remain underexplored and require systematic benchmarking.

Sustainability has also emerged as a key driver in adhesive research, in line with global green manufacturing goals. Conventional polymer adhesives are predominantly petroleum-derived and difficult to recycle, motivating efforts toward bio-based and recyclable systems. Renewable sources such as lignin, tannins, or plant-derived resins offer sustainable alternatives [203], though these often face trade-offs in mechanical strength, thermal stability, or processing windows. Recent work is addressing such limitations by tailoring molecular weight, crosslink density, and curing kinetics to optimize properties for industrial use. For example, ref. [204] demonstrated that lignin-based polyols derived from rice straw could replace fossil-based components in polyurethane hot-melt adhesives. While rheological and thermal properties were comparable to conventional systems, lap-shear strength remained slightly lower (~6–8 MPa vs. 8–10 MPa), highlighting the need to improve lignin reactivity and substrate compatibility. Despite this limitation, the valorization of agricultural by-products underscores the alignment of such systems with circular economy principles.

Chitosan-based formulations are also gaining traction, particularly in sustainable wood adhesives, due to their antimicrobial resistance and durability—properties often lacking in other bio-based systems. For instance, ref. [205] reported a biomass-derived polyester cross-linked with chitosan and amino trimethylene phosphonic acid, achieving shear strengths above 10 MPa, passing boiling-water resistance tests, and demonstrating additional features such as mildew inhibition and flame retardancy. These properties surpass multiple industry standards, showing that bio-based adhesives can meet—or even exceed—performance requirements for structural applications. Nevertheless, processing constraints remain: many bio-based adhesives require tight control over curing conditions (moisture, temperature) and often exhibit higher viscosity or longer cure times, limiting compatibility with high-throughput production.

A complementary innovation pathway is the design of reversible adhesives that enable the disassembly and recycling of composite structures at end-of-life. Examples include reversible polyurethanes with Diels–Alder crosslinks, which debond on demand at ~150 °C, facilitating repair or recycling of automotive body panels. Similarly, vitrimer-based epoxy systems have been adapted for electronics, enabling circuit board disassembly. These approaches directly address sustainability and waste management challenges while enabling repair-friendly and recyclable bonded structures.

Despite these promising directions, operational barriers remain. One persistent obstacle is curing time. Conventional thermosets often require hours of high-temperature curing, incompatible with the cycle times demanded in automotive or aerospace manufacturing. For instance, automotive production lines require cure times below five minutes, especially for battery pack assembly, driving the market for fast-curing epoxy adhesives. To optimize cure schedules, recent research [206] embedded single-walled carbon nanotube (SWCNT) networks into adhesives, enabling improved cure monitoring and post-cure optimization. This approach increased strength by ~60% and enhanced elongation. Nevertheless, widespread adoption is constrained by process variability: maintaining precise control of temperature, humidity, and cycle duration across large assemblies remains unresolved, and many adhesive systems still lack formulations compatible with industrial cycle times.

Another critical challenge is quality control and nondestructive inspection (NDI/NDT). Reliable detection of flaws such as voids, kissing bonds, incomplete curing, or interfacial debonding is essential for safety-critical joints in aerospace and automotive structures. Yet, there is currently no standardized inspection protocol. A recent review [3] highlighted the need for multi-method NDT approaches (ultrasonic, thermography, radiography) for defect detection in complex assemblies, particularly in aircraft joints where reliability is paramount. Efforts are underway to integrate smart sensors and automated NDT systems for faster and more accurate inspections, but challenges in cost, accessibility, and industrial workflow integration remain.

Computational modeling and machine learning (ML) represent another frontier in adhesive development. ML algorithms can leverage large datasets to predict adhesive performance under diverse conditions, accelerating the discovery of new formulations with tailored properties [207]. Frameworks now exist that predict critical adhesive parameters such as glass transition temperature, elastic modulus, and lap-shear strength directly from formulation descriptors [208]. By reducing reliance on trial-and-error experiments, ML-guided approaches drastically shorten development cycles. Surrogate ML models have also been employed to extract traction–separation laws (TSLs) directly from experimental load–displacement data [209,210], eliminating the need for repeated finite element calibration and reducing computational cost.

Beyond property prediction, ML has been combined with bioinspired and microstructured adhesive design. Deep-learning frameworks have been used to optimize fibril and pillar architectures, producing adhesive systems with empirically validated improvements in strength and load distribution efficiency [211]. This closed-loop integration of cohesive-zone modeling, experimental validation, and materials informatics has accelerated the design of next-generation adhesives with tailored fracture toughness, durability, and multifunctionality. For example, ref. [212] reported an AI-assisted stretchable bilayer hydrogel patch combining strong skin adhesion, mechanical flexibility, and reliable biosensing. The integration of random forest and CNN models enabled near-perfect predictive performance in monitoring sweat pH and glucose, validated against standard analytical methods. Similarly, ref. [213] applied neural network-based frameworks to optimize fibrillar adhesives inspired by gecko adhesion, predicting fibril compliance distributions that enhanced load sharing in complex geometries. These findings underscore the ability of AI-driven strategies to design advanced adhesives with properties not easily achievable through conventional methods.

In conclusion, the integration of nanotechnology, self-healing strategies, bio-based formulations, reversible systems, advanced processing methods, and AI-driven design represents a comprehensive roadmap for next-generation adhesive technologies. Despite progress, unresolved conflicts remain between sustainability and performance, healing efficiency and structural integrity, or processing speed and scalability. Bridging these gaps will require interdisciplinary approaches that combine experimental chemistry, computational modeling, and industrial-scale validation. The convergence of these directions holds the potential to transform adhesives into smarter, greener, and more reliable enablers of future composite technologies.

## 5. Conclusions

Polymer adhesives are indispensable in composite structures, functioning both as matrices in fiber-reinforced systems and as bonding agents in structural joints. Their lightweight, high-strength, and durable characteristics have secured their place in aerospace, automotive, marine, and civil engineering applications. This review has examined their mechanical performance—including tensile and shear strength, fracture toughness, fatigue, creep, and impact resistance—alongside experimental characterization techniques and advanced numerical tools such as cohesive zone models, finite element analysis, and molecular dynamics. Together, these approaches provide the foundation for understanding and improving adhesive performance across multiple scales.

Polymer adhesives in composite joints exhibit distinct mechanical responses under different loading conditions, each governed by a combination of adhesive chemistry, bond-line geometry, interfacial design, and environmental influences. In tensile loading, bond-line thickness and interfacial adhesion strongly influence stress distribution, where thinner layers provide high stiffness but risk premature failure, while thicker layers improve toughness at the expense of strength. Under shear conditions, load transfer efficiency is highly sensitive to bond-line uniformity, adherend stiffness, and surface preparation, with defects or poor adhesion accelerating debonding. Fracture behavior is dictated by mode mixity and crack path competition between cohesive and adhesive zones, with toughened epoxies and nanofillers delaying crack growth but posing challenges for predictive modeling. In fatigue loading, damage evolves gradually through microcrack initiation and propagation in the adhesive and interfacial regions, with cyclic creep, environmental degradation, and bond-line irregularities reducing lifetime; although cohesive zone and viscoelastic–damage models have advanced, their transferability across systems remains limited. Long-term creep and stress relaxation introduce additional complexity, as temperature, humidity, and aging accelerate stiffness reduction and time-dependent deformation, while classical linear viscoelastic models are insufficient to capture the multi-stage creep observed in real joints. Finally, under impact and high strain-rate loading, adhesives often transition from ductile to brittle responses, exhibiting enhanced strength and toughness with increasing rate but also undergoing abrupt shifts in failure mode; nanomodified and hybrid formulations show promise in improving dynamic toughness, though scalability to structural applications is not yet fully established.

Despite these advances, several persistent challenges limit the full exploitation of polymer adhesives. Optimizing bond-line thickness remains a delicate trade-off: thin layers promote efficient load transfer but amplify stress concentrations, while thicker layers enhance toughness but compromise shear strength. Durability under service conditions is another critical issue, as adhesives are vulnerable to thermal degradation, hydrolysis, plasticization, and chemical attack. Manufacturing-related factors—including surface preparation, curing control, and defect management—further complicate reliable large-scale implementation. Defects such as voids, incomplete curing, or kissing bonds are not always detectable with current inspection methods, underscoring the need for robust, standardized testing and non-destructive evaluation (NDE) protocols.

Future research is converging on several key directions. One is the development of nanomaterial-modified adhesives, where graphene, carbon nanotubes, or silica nanoparticles improve fracture toughness, crack resistance, and environmental durability. Another is the design of smart adhesives with self-healing functionality, based on reversible covalent bonding, supramolecular interactions, or encapsulated healing agents. These systems have shown high healing efficiencies in controlled studies, but their long-term performance under realistic service conditions remains an open question.

Sustainability is increasingly shaping adhesive innovation. Bio-based formulations derived from lignin, tannins, or chitosan present renewable alternatives to petroleum-based systems, while vitrimer- or Diels–Alder-based reversible adhesives offer recyclability and controlled disassembly. However, these systems often face trade-offs in cohesive strength, thermal stability, or processing ease. Achieving industrially viable performance while preserving recyclability and biodegradability is an ongoing challenge. Fast-curing adhesives are also in high demand, particularly for automotive manufacturing, where current thermoset systems remain incompatible with high-throughput production. Embedded nanonetworks and cure-monitoring sensors show potential to reduce cycle times and ensure more consistent bond quality, but industrial adoption is still limited.

Another major obstacle is quality assurance. NDE methods—ultrasonics, thermography, and radiography—are essential for detecting hidden defects in large, safety-critical joints, yet no single standardized protocol currently exists. The integration of automated NDE with digital twin approaches and smart sensors offers a promising way forward, though practical challenges related to cost and workflow integration persist.

Finally, computational modeling and artificial intelligence are poised to transform adhesive design and application. Machine learning (ML) frameworks can predict key properties such as glass transition temperature, modulus, and shear strength directly from compositional data, accelerating formulation discovery while reducing dependence on costly trial-and-error methods. Surrogate ML models can also provide traction–separation laws for cohesive zone models without extensive finite-element calibration. Beyond prediction, AI-driven optimization is enabling the design of fibrillar, bioinspired, and multifunctional adhesives with tailored architectures and enhanced performance, as well as stretchable, hydrogel-based systems for wearable devices. These developments illustrate the potential of AI-assisted approaches to establish a closed-loop discovery cycle, where experiments, modeling, and optimization are tightly integrated.

In conclusion, polymer adhesives remain a cornerstone of modern composite structures, combining mechanical performance with structural versatility. Yet their widespread adoption continues to be challenged by durability concerns, manufacturing limitations, and the absence of standardized testing and inspection protocols. Addressing these gaps requires interdisciplinary collaboration across polymer chemistry, materials science, mechanical engineering, and computational modeling. By leveraging nanotechnology, self-healing chemistries, bio-based alternatives, and AI-driven design, the next generation of adhesives can achieve not only enhanced performance and reliability but also alignment with global sustainability goals. Future innovations will enable adhesives that are stronger, smarter, faster-curing, and recyclable, ensuring their continued relevance in high-performance and environmentally responsible composite structures.

## Figures and Tables

**Table 2:** Overview of testing and analysis methods for polymer adhesives.

Category	Methods/Tests	Purpose, Key Insights, and Application to Composite–Adhesive Systems	References
4.1 Mechanical Testing Approaches	Tensile Testing (UTMs)	Measures tensile strength, modulus of elasticity, and elongation at break. In composite–adhesive joints, direct tensile tests are less common due to stress concentrations; results are mainly used to characterize bulk adhesive properties rather than joint performance. Generates stress–strain curves to assess load-bearing capacity and deformation behavior. Limitations: Stress concentrations often cause premature failure at the grips, making results less representative of in-joint performance.	[43]
	Shear Testing (Lap Shear: ASTM D1002, D5656)	Evaluates shear resistance, joint strength, and load transfer efficiency. ASTM D1002 [44] is most relevant for single-lap joints with thin adhesives (<0.25 mm) and is widely used for metal–composite bonding, whereas ASTM D5656 [45] uses thick adherends to minimize bending effects and is preferred for composite–composite joints. Failure mode analysis (cohesive vs. adhesive) helps optimize surface preparation and curing parameters. Limitations: Lap shear results are highly geometry-dependent, and peel stresses may overshadow shear, limiting their predictive capability for real joints.	[10,46]
	Compression Testing	Determines compressive strength, modulus, and failure strain. Important for bonded composite assemblies subjected to through-thickness compression or impact loads. Special fixturing is needed to avoid buckling of composite adherends. Limitations: Edge effects and buckling of adherends often interfere, making reproducibility a challenge.	[47,48]
4.2 Fracture and Fatigue Testing Methods	Mode I (DCB Test)	Assesses crack opening resistance (Mode I fracture toughness). Standardized by ASTM D5528 [49] for composite laminates; applicable to adhesively bonded joints when crack growth occurs along the bondline. Provides critical strain energy release rate GICG_{IC} used in CZM calibration. Limitations: Pre-crack insertion methods influence results, and fiber bridging in composites can artificially elevate toughness values.	[50,51]
	Mode II (ENF Test)	Evaluates shear-driven crack growth resistance. ASTM D7905 [52] is typically used; data is crucial for bonded composite joints under in-plane shear loading. Helps identify weak interfaces and interfacial toughness improvements after surface treatment. Limitations: Test setup is sensitive to loading asymmetry, and frictional effects at the crack tip can distort measured toughness.	[53,54]
	Mixed Mode Bending (MMB)	Combines Mode I and II loading to replicate real-world multiaxial stress states in joints. Useful for automotive/aerospace joints subjected to peel + shear; enables derivation of mixed-mode interaction criteria for CZM. Limitations: Accurate control of mode mixity is difficult; results are highly sensitive to fixture alignment.	[53,55]
	Fatigue Crack Growth Tests	Examines long-term durability under cyclic loads. ASTM D3479 [56] and D3166 [57] can be adapted for bonded joints; results reveal growth rates da/dN vs. ΔG, informing service-life predictions for composite structures. Limitations: Time-intensive testing, high scatter in fatigue data, and strong sensitivity to environmental conditions.	[58,59]
	Digital Image Correlation (DIC)	Non-contact optical technique for real-time strain mapping and crack growth monitoring. Particularly valuable for composite–adhesive joints, as it captures strain localization near the bondline and validates FE models under complex loading. Limitations: Requires high-quality surface preparation, is sensitive to lighting, and typically limited to surface strain fields rather than subsurface mechanisms.	[60,61]
4.3 Microscopic and Spectroscopic Failure Analysis	Scanning Electron Microscopy (SEM)	Examines fracture surfaces at high magnification. Used to distinguish cohesive, adhesive, and interfacial failures in composite joints; reveals fiber bridging, resin remnants, and failure initiation sites. Limitations: Requires destructive sample preparation and only provides post-mortem analysis.	[62,63]
	X-ray Computed Tomography (XCT)	Non-destructive 3D imaging to detect voids, porosity, and microcracks within adhesive layers. Especially relevant for thick composite joints where internal defects affect load transfer and fatigue resistance. Limitations: Limited spatial resolution for thin bondlines and relatively high cost/time per scan.	[64,65]
	Spectroscopy (FTIR, Raman)	Analyzes chemical composition, curing reactions, and degradation mechanisms. Useful for verifying surface treatments (e.g., plasma or primer presence), degree of cure, and tracking adhesive aging in service. Limitations: Typically requires small-scale samples; depth penetration is limited, making it difficult to assess bulk adhesive chemistry in joints.	[66,67]

**Table 3:** Typical tensile and shear strength ranges of adhesive systems [93,94,95,96,97,98,99,100].

Adhesive System	Tensile Strength (MPa)	Shear Strength (MPa)	Notes/Variability Sources
Epoxy	30–85	15–35	High strength and stiffness; brittleness remains an issue; toughening via rubber particles, thermoplastics, or nanofillers (graphene, alumina) significantly improves toughness; curing time and temperature strongly influence performance.
Polyurethane	14–92	7–25	Wide variability: conventional PU adhesives show low strength (~14 MPa); advanced Diels–Alder cross-linked PU and disulfide-bonded PU report tensile strengths equal to or surpassing epoxies (60–92 MPa). Highly flexible, good for dynamic and harsh environments.
Acrylics	20–55	10–25	Moderate strength, relatively easy to use; toughness and performance highly dependent on curing conditions and substrates; bridge gap between strength and processability.
PEEK (Polyether ether ketone)	70–100	20–40	High-performance thermoplastic with excellent mechanical stability; often used as a matrix or surface-modified for adhesion; adhesion strength depends strongly on surface treatment (plasma, chemical etching).
Nylon (Polyamide)	40–80	15–30	Strong hydrogen bonding capacity but moisture sensitivity reduces performance; adhesion often enhanced via surface functionalization or coupling agents. Used as matrix in fiber composites or as adhesive in structural plastics.

**Table 4:** The typical fracture toughness ranges by adhesive system [6,117,118,119,120].

Adhesive System	Typical GIc Range (J/m^2^)	Typical GIIc Range (J/m^2^)	Remarks
Epoxy	200–800 (toughened: up to 1500)	1000–2500	Aerospace/automotive standard; toughened epoxies with rubber/nanofillers achieve very high toughness
Polyurethane (PU)	300–1200	1500–3000	High toughness and flexibility; excellent under impact and fatigue loading
Acrylics (MMA, Cyanoacrylate, etc.)	200–600 (toughened: up to ~1000)	800–2000	Fast-curing structural systems; unmodified forms brittle, but toughened grades show significant improvement
PEEK-based adhesives	400–1200	1200–2500	High-performance thermoplastic adhesives; retain toughness at elevated temperatures
Nylon-based adhesives	300–900	1000–2200	Good toughness and flexibility; sensitive to moisture uptake, which may affect long-term durability

## Data Availability

No new data were created or analyzed in this study.
